# Development of an inhibiting antibody against equine interleukin 5 to treat insect bite hypersensitivity of horses

**DOI:** 10.1038/s41598-023-31173-y

**Published:** 2023-03-10

**Authors:** Nora Langreder, Dorina Schäckermann, Doris Meier, Marlies Becker, Maren Schubert, Stefan Dübel, Thomas Reinard, Stefanie Figge-Wegener, Kristine Roßbach, Wolfgang Bäumer, Simone Ladel, Michael Hust

**Affiliations:** 1 Institut für Biochemie, Biotechnologie und Bioinformatik, Technische Universität Braunschweig, Spielmannstr. 7, 38106 Braunschweig, Germany; 2 Institut für Pflanzengenetik Abt II, Leibniz Universität Hannover, Herrenhäuser Straße 2, 30419 Hannover, Germany; 3 Novihum Technologies GmbH, Weidenstraße 70-72, 44147 Dortmund, Germany; 4 Wirtschaftsgenossenschaft deutscher Tierärzte eG (WDT), Siemensstraße 14, 30827 Garbsen, Germany; 5 Institut für Pharmakologie und Toxikologie, Fachbereich Veterinärmedizin, Freie Universität Berlin, Koserstraße 20, 14195 Berlin, Germany

**Keywords:** Applied immunology, Inflammatory diseases, Biotechnology, Biologics, Antibody therapy

## Abstract

Insect bite hypersensitivity (IBH) is the most common allergic skin disease of horses. It is caused by insect bites of the *Culicoides* spp. which mediate a type I/IVb allergy with strong involvement of eosinophil cells. No specific treatment option is available so far. One concept could be the use of a therapeutic antibody targeting equine interleukin 5, the main activator and regulator of eosinophils. Therefore, antibodies were selected by phage display using the naïve human antibody gene libraries HAL9/10, tested in a cellular in vitro inhibition assay and subjected to an in vitro affinity maturation. In total, 28 antibodies were selected by phage display out of which eleven have been found to be inhibiting in the final format as chimeric immunoglobulin G with equine constant domains. The two most promising candidates were further improved by in vitro affinity maturation up to factor 2.5 regarding their binding activity and up to factor 2.0 regarding their inhibition effect. The final antibody named NOL226-2-D10 showed a strong inhibition of the interleukin 5 binding to its receptor (IC_50_ = 4 nM). Furthermore, a nanomolar binding activity (EC_50_ = 8.8 nM), stable behavior and satisfactory producibility were demonstrated. This antibody is an excellent candidate for in vivo studies for the treatment of equine IBH.

## Introduction

Insect bite hypersensitivity (IBH), also called summer eczema, is the most common allergic skin disease of horses^1,2^. It is a chronical relapsing seasonal disease caused by insect bites of the *Culicoides* spp.^3,4^. The prevalence, strongly correlating with the distribution of the *Culicoides*, ranges worldwide from 3% to 60%^5^. In particular, Icelandic horses that are imported from Iceland to e.g. Europe are affected with a prevalence of > 50% due to the fact that *Culicoides* spp. do not exist in Iceland^5,6^. Symptoms usually occur from spring until autumn depending on the mosquito flight season^7^. Affected horses suffer from hair loss, skin lesions, strong pruritus and possibly secondary infections, typically at the mane and tail and along the dorsal and ventral midline^4,8^.

So far, there is no effective treatment option that can be applied long term without safety concerns. Usually, exposure to the insects is avoided by stabling the horses, covering them with rugs or using insect repellents. Symptomatic treatment with glucocorticoids can have strong side effect^9^ and application of a histamine receptor 1 antagonist failed to show efficacy^10^. Alternatively, allergen specific immunotherapy (ASIT) is a promising approach, but still needs further development and lacks well-defined allergens that are in this case a prerequisite for effective treatment^4,11^.

Different salivary gland proteins of the *Culicoides* spp. act as allergens causing a type I allergy with involvement of a type IVb allergy^4,5,11,12^. In type I allergic reactions, T cells polarize into CD4^+^ T_H_2 cells that secrete interleukin (IL)-4 and IL-13. These interleukins trigger a B cell class switch towards immunoglobulin E (IgE) producing plasma cells. The IgE antibodies bind to the FcεRI on mast cells and basophils. A re-exposure to the allergens leads to cross-linking of IgE molecules and thus a degranulation of the cells and subsequent release of inflammatory mediators like histamine and leukotrienes. In addition, chemokines and cytokines are released that cause the recruitment of effector cells like eosinophils, T_H_2 cells and basophils to the allergic site. In the late phase of type I allergies and in type IVb allergies, T_H_2 cells produce IL-5 which is responsible for the differentiation, activation, survival and chemotaxis of eosinophil cells and in this way enhances the allergic reaction^4,11,13^.

Eosinophil cells play a crucial role in the pathogenesis of equine IBH. Besides an infiltration of eosinophils to the allergic site, a correlation of the amount of blood eosinophils and severity of IBH has been described^8^. IL-5 is the main activator and regulator of blood and tissue eosinophil cells^14^. Therefore, targeting equine IL-5 (eqIL-5) is a promising way to inhibit the allergic reaction caused by eosinophilia and thereby treating equine IBH. For humans, there are already two approved antibodies against IL-5 (Mepolizumab and Reslizumab) and one antibody against the IL-5 receptor alpha subunit (Benralizumab) for the treatment of eosinophilic asthma. All three antibodies prevent the binding of IL-5 to its receptor and were found to be effective and safe^15,16^.

Another approach also targeting eqIL-5 to treat IBH of horses has been published recently^8,17,18^. There, the researchers developed an active vaccination that contains the interleukin linked to a virus-like particle (VLP) which induced the generation of neutralizing antibodies. According to the authors, their treatment resulted in a reduction of the symptoms. In addition, a decrease of circulating eosinophils after first and second year treatment and a reduced basophil count only after second year treatment was reported. Also, they reported no safety issues during their studies, in particular no difference in parasite presence prior and post vaccination during first and second year studies^17,18^.

Despite promising results and reversible antibody titers, we assume there might be a potential risk of unpredictable future long-term consequences when autoantibodies against the body’s own interleukin are generated by active vaccination. As the main regulator of eosinophil cells, IL-5 plays a significant role in the protective immune response against invading pathogens like helminths, virus and bacteria^14,19^. Also, antibodies generated by active vaccination could potentially enhance the allergic reaction by recruiting more immune cells due to the effector function mediated by the fragment crystallizable (Fc) region.

Therefore, we consider a passive vaccination with a well-defined neutralizing monoclonal antibody as a safer option. Immunoglobulin G (IgG) antibodies have a half-life of approximately 21 days and would consequently need to be administered regularly over the summer period. This has the advantage that the treatment is better controllable and could be stopped at any time point if undesired immune reactions occurred. In order to avoid any unwanted immune reaction, we generate antibodies with all constant parts as equine domains, only the heavy chain variable domain (VH) and light chain variable domain (VL) are still of human origin. We choose the IgG6 subclass which was described to have no Fc-mediated effector function^20^, so an additional recruitment of immune cells to the allergic site is avoided.

In the veterinary field, few therapeutic antibodies have already been approved for treatment of dogs and cats^21–23^. In comparison, horses have a significantly higher body weight which implies the challenge of high production costs for recurring vaccinations during the summer season. We are aware that in the long term, a low-cost production system is required for the treatment to be economical.

In this report, however, we focus on the development of a potential antibody candidate for the therapeutic treatment of equine IBH. Such a therapeutic antibody must fulfill certain requirements depending on the application. In our case, we want to reduce the allergic reaction by selecting an antibody without effector function that prevents the binding of eqIL-5 to its receptor. Our main criterion for antibody selection is the inhibition effect. In this regard, specific antibody binding to the target with high binding affinity is also required. In addition to a strong inhibition, antibody stability is a necessity and a high producibility is advantageous.

For the antibody development, we first produced the antigen eqIL-5 recombinantly. Then, binding antibodies were selected by antibody phage display. Selected antibodies were tested for their functionality in a cellular in vitro inhibition assay. The most promising candidates were characterized with regard to their stability and specificity and then subjected to an in vitro affinity maturation for further improvement of their inhibition effect.

## Results

### Production of recombinant eqIL-5

Target availability is a prerequisite for the development of a therapeutic antibody. In this study, the target eqIL-5 was produced recombinantly. It consists of 134 amino acids, of which the first 19 amino acids make up the signal peptide sequence (Uniprot O02699). EqIL-5 is a disulfide-linked homodimer^24^ with a size of about 26 kDa. The interleukin with an 8 × His-tag was successfully produced in Expi293F suspension cells (Thermo Fischer Scientific) and purified by nickel-loaded Sepharose, eluted with 250 mM Imidazole and dialyzed in 1 × PBS (Supplementary Fig. S1).

### Selection of antibodies against eqIL-5 via antibody phage display

The next step of the development of a therapeutic antibody against eqIL-5 was the selection of antibodies that bind to this target via antibody phage display. In this study, three different panning approaches were applied in order to select a large number of diverse binders: Firstly, panning in a multititer plate (MTP) with immobilized antigen^25^, secondly, panning in solution with biotinylated antigen^26^ and thirdly, capture panning with human Fc-tagged antigen. For all three methods the human naïve antibody gene libraries HAL9 (lambda) and HAL10 (kappa) were used as starting material^27^. Table 1 summarizes the number of individual binders selected by the three panning strategies in relation to the number of clones that was tested in the screening ELISA. With all three panning techniques, individual binders could be selected against the target eqIL-5. The average hit rate for panning in MTP was 2.0%, for panning in solution 4.1% and for capture panning 1.1%. 29 lambda binders and 7 kappa binders were selected, which results in an overall hit rate of 3.9% for the HAL9 and 1.1% for the HAL10 library. In total, 36 binders were selected against eqIL-5. One of these binders (NOL46-1-A1) was selected using all three different techniques, thereby reducing the number of unique binders to 34. In a next step, these antibodies were cloned and produced in the human single chain fragment variable (scFv)-Fc format for further analysis. Six antibodies could not be produced in this format and were therefore excluded from further analysis. This resulted in 28 remaining scFv-hFc antibodies against eqIL-5 for a first functional screening.

**Table 1:** Selection of unique binders against eqIL-5 via different antibody phage display techniques.

Library	Panning in MTP	Panning in solution	Capture panning
HAL9	12/368	15/276	2/92
HAL10	3/368	4/184	0/92

### Selection of lead candidates with a cellular in vitro inhibition assay

In the next step, the binding antibodies were tested regarding their functionality. For this purpose, a cellular inhibition assay was established in order to test whether the binding of the selected antibodies to the antigen inhibits the interaction of the interleukin and its receptor on a cell surface. Expi293F suspension cells were transfected transiently with the eqIL-5 receptor DNA subunits IL5RA (Uniprot A0A3Q2L5Z7) and CSF2RB (Uniprot F7DHE0) and co-transfected with eGFP (GenBank AEI54555.1) to differentiate transfected from non-transfected cells. The binding of the interleukin to its receptor on the cells was detected via the 8 × His-tag and cells were analyzed by flow cytometry (Supplementary Fig. S2).

**Figure 1:**
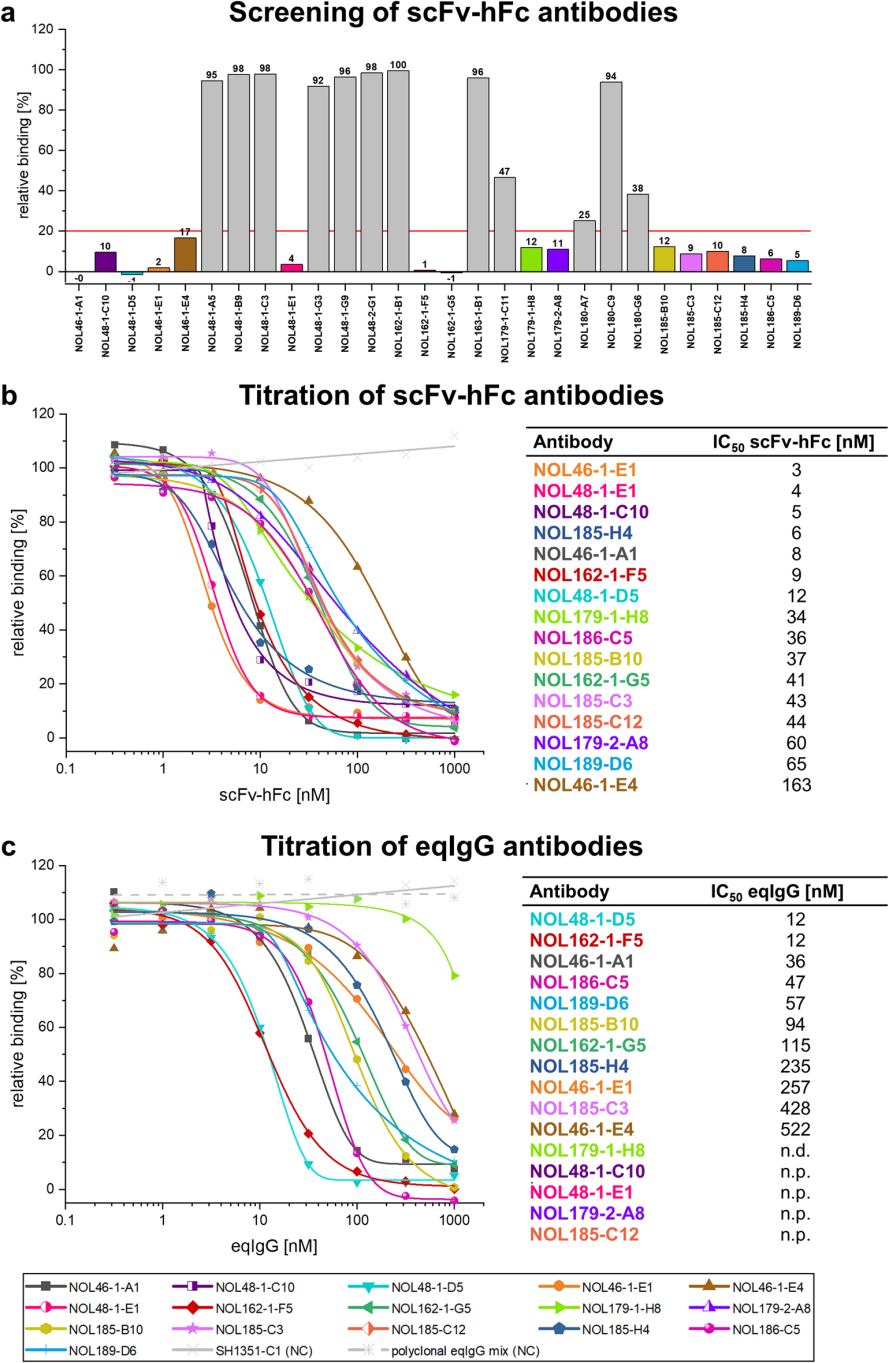
Cellular inhibition assay. (**a**) Screening inhibition assay with 28 scFv-hFc antibodies against eqIL-5 on receptor positive cells using 1000 nM antibody and 5 nM interleukin (related to dimer) (molar ratio 200:1). The cut-off for the selection of inhibiting antibodies is marked with a red line and antibodies for further analysis are marked in colors. (**b,c**) Titration inhibition assay with antibodies in (**b**) scFv-hFc format and (**c**) eqIgG6 format using 1000 nM–0.32 nM antibody and 5 nM antigen (related to dimer) (molar ratio 200:1–0.064:1). IC_50_ values were determined with OriginPro using the Logistic5 Fit. For the isotype control a linear fit was applied. Screening and titration assays were performed in single measurements (n = 1). *n.p.* not producible, *n.d.* not determined.

Figure 1 presents the relative binding of the interleukin to the cell after pre-incubation with the individual antibody. As a reference for 100% binding, antigen binding to the cell without application of test antibody was measured. The selected antibodies in scFv-hFc format were screened in the cellular inhibition assay using 1000 nM antibody and 5 nM antigen (molar ratio 200:1) (Fig. 1a). The cut-off for inhibiting antibodies was set at 20% so that only antibodies with a significant inhibition effect were considered for next steps. 16 out of the 28 antibodies remained below this cut-off. Nine of these antibodies were selected by panning in MTP, five by panning in solution and one by capture panning. Additionally, the antibody NOL46-1-A1 selected with all three panning strategies was one of the best three inhibiting antibodies. To further characterize the inhibition effect of these 16 antibodies, they were tested in concentrations ranging from 1000 nM to 0.32 nM with a constant antigen concentration of 5 nM (related to dimer) (molar ratio 200:1–0.064:1). Here, a comparison was made for the antibodies in the scFv-hFc format (Fig. 1b) and the final equine IgG6 (eqIgG6) format (Fig. 1c). For the eqIgG6 format all constant domains of the antibodies were replaced with equine domains (IGHC IMGC000040, IGLC^28^, IGKC IMGT000053), only the VH and VL remained of human origin. The antibodies NOL48-1-C10, NOL48-1-E1, NOL179-2-A8 and NOL185-C12 could not be produced as eqIgG6 and were therefore excluded from further selection. In both formats an unrelated isotype control (SH1351-C1 anti N-cadherin propeptide) was included.

In order to compare the inhibition effect, the antibody concentration necessary to reduce the relative binding to 50% (IC_50_) was determined. The tables presented in Fig. 1b and c summarize the IC_50_ values for the selected antibodies in scFv-hFc and eqIgG6 format. IC_50_ values for the scFv-hFc antibodies ranged from 3 nM (NOL46-1-E1) to 163 nM (NOL46-1-E4) and for eqIgG6 antibodies from 12 nM (NOL48-1-D5 and NOL162-1-F5) to 522 nM (NOL46-1-E4). The IC_50_ value for NOL179-1-H8 as eqIgG6 could not be determined due to its low inhibition effect.

Nearly all tested scFv-hFc antibodies had lower IC_50_ values compared to the corresponding antibody in eqIgG6 format. To gain a better understanding of this phenomenon, two antibodies (NOL46-1-A1 and NOL46-1-E4) were compared as scFv-hFc, scFv-eqFc and eqIgG (schmatic illustration of the antibody formats presented in Supplementary Fig. S3) in this assay. Antibodies in the equine scFv-Fc and human scFv-Fc format showed a comparable inhibition effect, which was different from the inhibition effect of the eqIgG antibodies (Supplementary Fig. S4a). This indicates that the antibody format but not the Fc-part is responsible for the different inhibition behavior. Interestingly, despite the better inhibition effect in the scFv-Fc format, the same antibodies (NOL46-1-A1 and NOL46-1-E4) presented a lower binding activity as scFv-eqFc compared to the corresponding eqIgG in titration ELISA (Supplementary Fig. S4b and c).

The results show a better inhibition of the scFv-Fc format compared to the IgG format. However, out of the 16 antibodies only NOL46-1-A1 and NOL46-1-E4 were producible in the scFv-eqFc format and this only in small amounts (5 mg/L and 9 mg/L). Therefore, it was not reasonable to focus on antibodies in scFv-eqFc format during the development process. Furthermore, the full IgG is the preferred format for therapeutic applications since it is most established in therapeutic and regulatory affairs. Additionally, the Fab fragments of an IgG ususally lead to higher stability than the scFv fragments of an scFv-Fc^29^ and an IgG has a tendency towards a a longer half-life compared to an scFv-Fc^30^.

Hence, the selection of lead candidates for further development was based on the inhibition of the antibodies as eqIgG6. The two selected eqIgG6 antibodies were NOL48-1-D5 (selected by panning in solution) and NOL162-1-F5 (selected by panning in MTP), both with an IC_50_ of 12 nM (molar ratio of antibody (bivalent) to antigen (dimer): 2.4).

Besides binding and inhibition, stability and specificity are also of great importance for an antibody for therapeutic application. Before further experiments were performed, these characteristics needed to be confirmed in order to enhance the chances of obtaining final candidates with the desired features. Thus, the two antibodies NOL48-1-D5 and NOL162-1-F5 were characterized in the eqIgG6 format regarding their stability in titration ELISA, inhibition assay and size exclusion chromatography (SEC) (Supplementary Fig. S5) and regarding their specificity in an unspecificity ELISA (Supplementary Fig. S6). In summary, only slight decrease of stability was detected when antibodies were stored up to 28 days at 37 °C at a concentration of 0.3 mg/mL. Also, no unspecific behavior of the antibodies was observed in the unspecificity ELISA.

### In vitro affinity maturation for improvement of lead candidates

**Figure 2:**
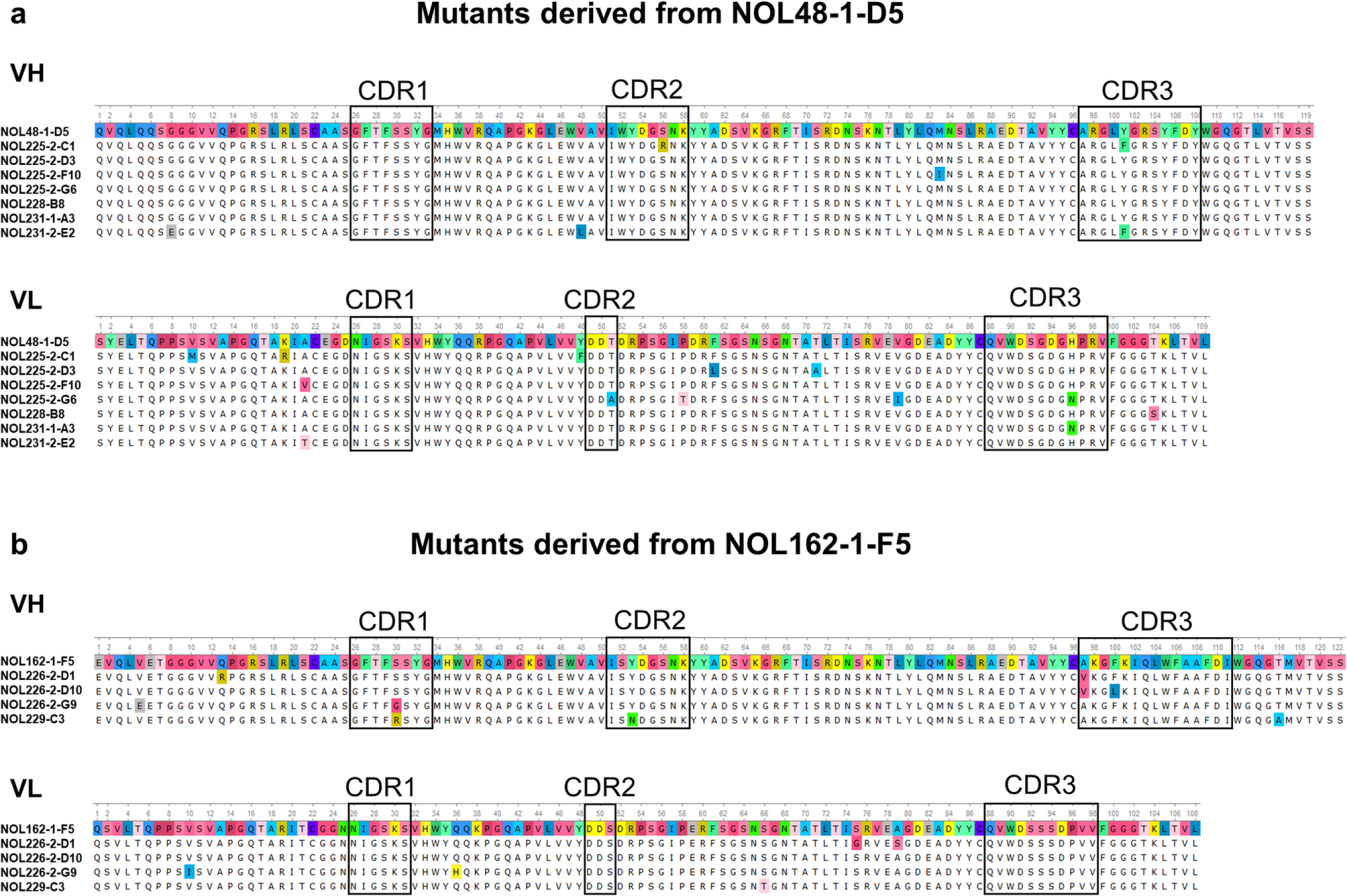
Sequence alignment of affinity-matured antibodies. (**a**) Alignment of the VH and VL region of mutants derived from NOL48-1-D5. (**b**) Alignment of the VH and VL region of mutants derived from NOL162-1-F5. The CDR regions are marked in boxes. The parental sequences are set as references and amino acid substitutions in other sequences are marked in color. Alignments were constructed using Unipro UGENE.

Next, an in vitro affinity maturation was performed with the lead candidates in order to improve their binding activity and consequently their inhibition effect. Within the scope of library generation from a single parental sequence many approaches have been developed for the introduction of random mutations at the nucleotide level, e.g. error prone PCR^31^, mutator bacterial strains^32^ and site-specific mutagenesis of the complementary determining regions^33^. Here, we chose several rounds of error-prone PCR to generate scFv mutagenesis libraries. With these scFv mutagenesis libraries a panning approach with low amount of antigen and extended washing and incubation steps was performed in order to select antibodies with improved binding activity. A screening ELISA with scFv supernatant was used to find clones with the highest ELISA signals. In addition, it was confirmed by Western Blot that the signals were not significantly biased by the producibility of the scFvs. In total, 3.8% of mutants derived from NOL48-1-D5 had increased signals compared to their parental scFv (up to factor 1.5). Regarding the mutants derived from NOL162-1-F5 there were 6.6% of hits with increased signals (up to factor 2.6). Seven mutants derived from NOL48-1-D5 and four mutants derived from NOL162-1-F5 were selected, all with a signal increase > 1.3 × compared to the corresponding parental scFv. An amino acid sequence alignment of the VH and VL of these affinity-matured antibodies is presented in Fig. 2.

The number of mutations at the amino acid level in the whole scFv region ranged for mutants derived from NOL48-1-D5 from 1 to 5 with an average of 2.7 amino acid exchanges (Fig. 2a). Mutants derived from NOL162-1-F5 had 2 to 4 amino acid exchanges with an average of 3.5 (Fig. 2b). Overall, the position of the mutations was spread over the whole sequences with no significant accumulation in the CDR regions. Only single positions of amino acid substitutions were found in several affinity-matured antibodies (NOL48-1-D5: VH at position 101, VL at position 21 and 96; NOL162-1-F5: VH at position 30 and 97).

**Figure 3:**
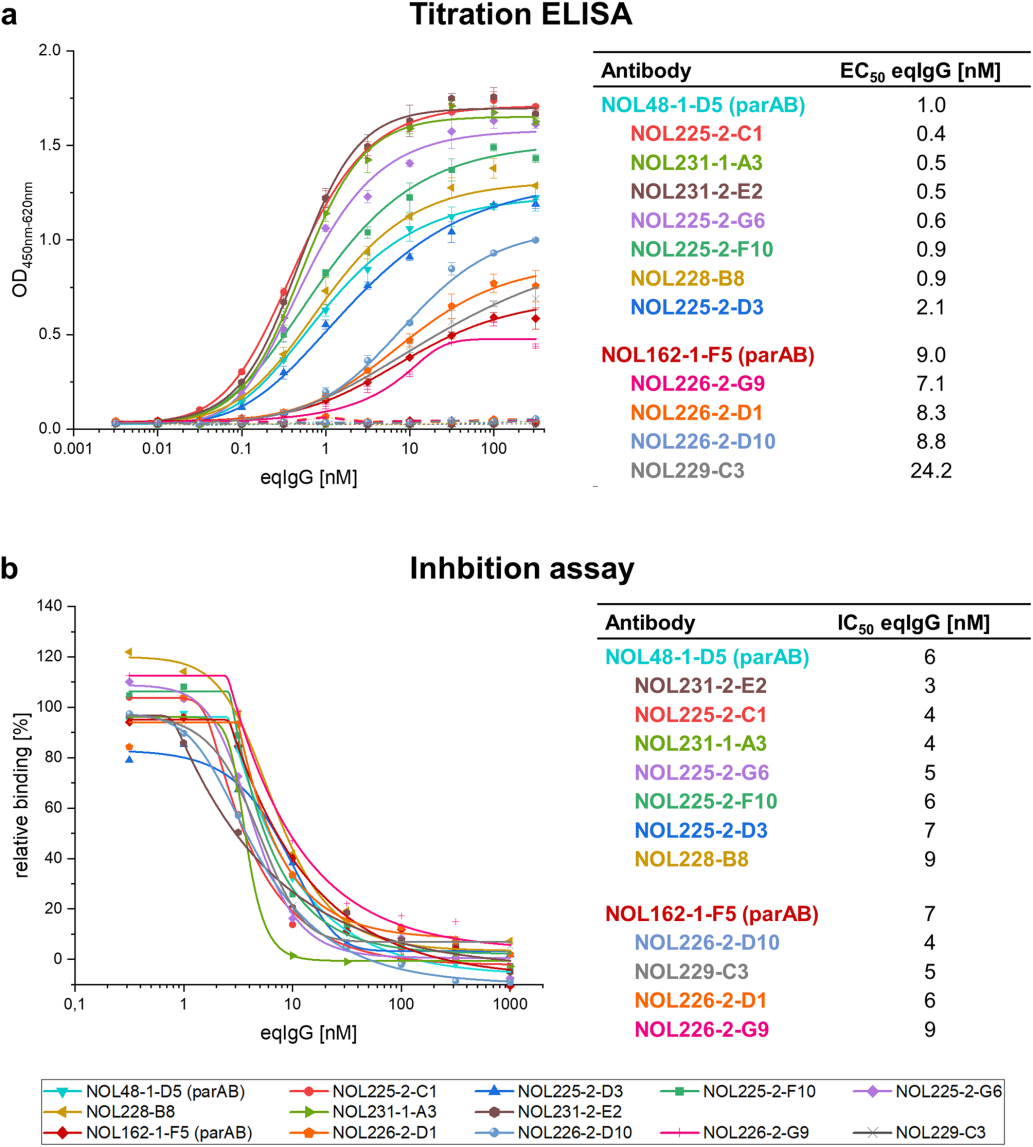
Titration ELISA and cellular inhibition assay for affinity-matured antibodies. (**a**) Titration ELISA of affinity-matured antibodies derived from the parental antibodies NOL48-1-D5 and NOL162-1-F5. Antibodies were titrated on immobilized antigen in eqIgG6 format using 316 nM–0.0032 nM antibody. As negative control, antibodies were titrated on 1% BSA (marked with dashed lines). EC_50_ values were determined with OriginPro using the Logistic5 Fit. Measurements were performed in triplicates (n = 3). (**b**) Titration inhibition assay with affinity-matured antibodies derived from the parental antibodies NOL48-1-D5 and NOL162-1-F5. Antibodies were titrated in eqIgG6 format using 1000 nM–0.32 nM antibody and 5 nM antigen (related to dimer) (molar ratio 200:1–0.064:1). IC_50_ values were determined with OriginPro using the Logistic5 Fit. Measurements were performed in single measurements (n = 1). *parAB* parental antibody.

The antibody mutants were cloned and produced as equine IgG6 antibodies in Expi293F suspension cells for final characterization. The affinity-matured antibodies were analyzed by titration ELISA (Fig. 3a) and cellular inhibition assay (Fig. 3b) including the EC_50_ and IC_50_ values in order to compare their binding activity and inhibition effect.

In the titration ELISA all tested antibodies bound specifically to eqIL-5 and not to BSA. Overall, the antibodies derived from NOL48-1-D5 had a stronger binding activity with EC_50_ values between 0.4 nM and 2.1 nM compared to the antibodies derived from NOL162-1-F5 with EC_50_ values ranging from 7.1 nM to 24.2 nM. Both parental antibodies belonged to the least affine antibodies compared to their respective mutants, only single mutants showed weaker binding. The affinity maturation improved the EC_50_ for NOL48-1-D5 from 1.0 nM to 0.4 nM (factor 2.5) and for NOL162-1-F5 from 9.0 nM to 7.1 nM (factor 1.3).

In the cellular inhibition assay the IC_50_ values ranged from 3 nM to 9 nM including all tested antibodies. Most mutants had a reduced IC_50_ value compared to both parental antibodies. The affinity maturation improved the IC_50_ value for NOL48-1-D5 from 6 nM to 3 nM (factor 2.0) and for NOL162-1-F5 from 7 nM to 4 nM (factor 1.8). Mutants derived from the different parental antibodies against eqIL-5 were similarly effective in the inhibition assay, despite different binding behavior in the titration ELISA. This could be explained by the assumption that the antibodies recognize different epitopes. In conclusion, the weaker binding in ELISA of mutants derived from NOL162-1-F5 is no criterion for exclusion since the main aspect for selection of final antibodies is the functionality tested in the cellular inhibition assay.

Many mutants were in a similar range regarding their inhibition effect. Considering the parameters IC_50_ value, curve progression and producibility of the antibodies, we chose NOL226-2-D10 (IC_50_: 4 nM; producibility: ~ 70 mg/L) and NOL231-1-A3 (IC_50_: 4 nM; producibility: ~ 30 mg/L) as most promising candidates. Further mutants, such as NOL225-2-C1 (IC_50_: 4 nM; producibility: ~ 15 mg/L) and NOL231-2-E2 (IC_50_: 3 nM; producibility: ~ 25 mg/L), could be considered as potential backup candidates.

The two antibodies NOL226-2-D10 and NOL231-1-A3 were produced in a 250 mL scale in Expi293F suspension cells. Here, the antibody NOL231-1-A3 was not stable in concentrations > 0.8 mg/mL. The antibody solution showed turbidity indicating that aggregation of the antibody occurred. Also, the antibody concentration measured spectrophotometrically was decreasing over time. Attempts to stabilize NOL231-1-A3 by changing the pH value were not successful. Therefore, NOL226-2-D10 was chosen as final candidate for further studies.

### Biochemical analysis of final candidate NOL226-2-D10

NOL226-2-D10 was analyzed in regard of its stability and specificity (Supplementary Fig. 7). The long-term stability of NOL226-2-D10 was examined in titration ELSA and cellular inhibition assay with samples stored at 4 °C to reflect storage conditions at final application. NOL226-2-D10 presented hardly any loss of binding activity (Supplementary Fig. S7a) and only slight decrease of the inhibition potential (Supplementary Fig. S7b) after being stored for up to 6 months at a concentration of 1.86 mg/mL at 4 °C. Also, no formation of aggregates or fragments was observed with samples being stored at a concentration of 1.86 mg/mL for 1 month at 4 °C or 2 months at 21 °C (Supplementary Fig. S7c). Furthermore, the unspecificity ELISA displayed no unspecific binding to any of the tested antigens (Supplementary Fig. S7d).

Additionally, the final antibody NOL226-2-D10 was tested in respect of its competitive inhibition potential in the in vitro inhibition assay, i.e. the antibody was not pre-incubated with eqIL-5, but applied to the eqIL-5 receptor expressing cells before adding eqIL-5. NOL226-2-D10 was still well-inhibiting with only a slight reduction of the inhibition effect (IC_50_ = 9 nM in the competitive inhibition assay compared to 4 nM in the original inhibition assay). In comparison, also the parental antibody NOL162-1-F5 was tested in the competitive inhibition assay and showed a reduction of its inhibition potential in the same ratio (IC_50_ = 15 nM in the competitive inhibition assay compared to 7 nM in the original inhibition assay) (Supplementary Fig. S8).

In summary, the lead candidate NOL226-2-D10 provides all required properties for further clinical development: A strong inhibition effect (IC_50_ = 4 nM, in competitive assay IC_50_ = 9 nM), nanomolar binding activity to the target (EC_50_ = 8.8 nM), satisfactory producibility (~ 70 mg/L in transient expression system), high stability and no indication for unspecific behavior.

## Discussion

IBH is the most common allergic skin disease of horses with no specific treatment option so far^4^. In this study, we developed an antibody against eqIL-5 that is a promising candidate for potential treatment of this disease.

In total, 36 binding antibodies to eqIL-5 were selected by antibody phage display from the human naïve antibody gene libraries HAL9/10^27^. Here, we compared three different panning procedures: panning in MTP with directly immobilized antigen, panning in solution with biotinylated antigen and capture panning with human Fc-tagged antigen. Compared to the panning in MTP the panning in solution is slightly more complex but allows the target to be present in its native form which can lead to improved epitope accessibility^26,34^. The protocol with captured antigen is comparable to the protocol with directly immobilized antigen but potentially allows better target accessibility. A drawback could be that the antigen is dimerized due to the coupling of two Fc-parts and therefore not in its original form. We could select antibodies with all three strategies indicating that a combination of panning strategies can increase the number and diversity of binders. In total, we selected 29 binders from the HAL9 library (lambda) and only seven binders from the HAL10 library (kappa). The higher yield of binders from the lambda library has been described before and may be caused by a better expression level of lambda scFvs compared to kappa scFvs in *E. coli*^35,36^. One binder (NOL46-1-A1) was selected using all three techniques, possibly explained by high producibility as scFv antibody fragment in *E. coli* and strong binding to a well accessible epitope of the antigen. Six of the 36 binders were not producible in the scFv-hFc format and were therefore excluded from further analysis.

A cellular in vitro inhibition assay was established for the selection of inhibiting antibodies. Overall, this assay provides a way to easily analyze the antibodies’ functionality in vitro when no native cell line expressing the desired receptor is availiable. The same assay setup was for example used successfully with ACE2 receptor expressing cells for a selection of inhibiting antibodies against SARS-CoV-2 spike protein^37,38^. Nevertheless, it has to be kept in mind that this assay is dependent on the state of the cells and their transfection efficiency. It was observed that absolute values could fluctuate on different measurement dates but the ranking of the antibodies within one measurement was always reproducible and therefore reliable for selection of the best inhibiting antibodies. 16 out of the 28 scFv-hFc antibodies (57%) reduced the binding of eqIL-5 to its receptor on the cell surface to less than 20%. For comparison, 13% of antibodies selected against SARS-CoV-2 RBD from the same antibody gene libraries HAL9/10 inhibited the interaction of S1-S2 with the ACE2 receptor to less than 25% in a similar cellular inhibition assay^37^. This high rate of inhibiting antibodies indicates that epitopes with crucial amino acids for the interaction of eqIL-5 with the receptor (described for human IL-5 by Patine et al.^24^) were well accessible during the panning procedure. Nine of the inhibiting antibodies derived from the panning in MTP, five from the panning in solution and one from the capture panning. This shows that important epitopes were still accessible even when the antigen was immobilized directly. Additionally, the antibody NOL46-1-A1 selected with all three panning strategies was one of the best inhibiting antibodies.

In the inhibition assay we observed that scFv-Fc antibodies showed stronger inhibition than IgG antibodies. Interestingly, the comparison of two antibodies in scFv-eqFc and eqIgG format showed a better inhibition effect of the scFv-eqFc antibody compared to the corresponding eqIgG antibody while the binding activity determined by titration ELISA was lower for the scFv-eqFc than for the eqIgG. A change of the affinity and behavior in functional assays is possible due to format conversion from scFv-Fc to IgG and individual for each antibody as already shown by others^37,39^.

The two antibodies that performed best as eqIgG6 in the inhibition assay, NOL48-1-D5 and NOL162-1-F5, were used for an in vitro affinity maturation. The affinity maturation improved the binding activity (EC_50_) in titration ELISA up to factor 2.5 for NOL48-1-D5 and up to factor 1.3 for NOL162-1-F5. For some antibodies, e.g. NOL226-2-D10, the titration curve seemed to be improved without significant reduction of the EC_50_ value compared to the parental antibody. This is due to the dependence of the EC_50_ on the maximum signal which was also increased in that case. In the cellular inhibition assay the inhibition effect (IC_50_) was improved up to factor 2.0 for NOL48-1-D5 and up to factor 1.8 for NOL162-1-F5. These improvements are relatively small compared to other reports which describe an increase of affinities for scFv antibodies by in vitro affinity maturation up to 100x^40^ or even 500x^41^. Nevertheless, in our case we consider the aforementioned decreases of the EC_50_ and IC_50_ values as significant and successful since the parental antibodies had already EC_50_ values in the nanomolar range and were well-inhibiting, leaving only small room for improvement. The mutants NOL226-2-D10 and NOL231-1-A3 had an IC_50_ value of 4 nM showing that only a molar ratio of antibody (bivalent) to antigen (dimer) of 0.8 was necessary to reduce the binding of the interleukin to its receptor to 50%. We hypothesize that the application of these antibodies in the horse would greatly reduce eqIL-5-derived eosinophilia but, depending on the applied dose, would not abolish the complete interaction of all eqIL-5 molecules with the receptor expressing cells. Dose finding in vivo will be required because in the context of equine IBH it may not be necessary to completely neutralize the binding of eqIL-5 to its receptor. It could be sufficient or even beneficial to only partially prevent binding so that natural functions in the body are still maintained. IL-5 is the main activator and regulator of blood and tissue eosinophil cells^14^. These cells play a crucial role in the protection against invading pathogens like helminths, virus and bacteria^19^. Therefore, a passive vaccination with monoclonal antibodies that have a half-life of approximately 3 weeks seems to be a safe treatment option in order to avoid an unpredictable impact on the protective immune response in the long term.

Despite successful improvement of the binding activity and inhibition effect due to the in vitro affinity maturation, we observed stability issues with one of the top candidates NOL231-1-A3. Only one amino acid substitution located in the CDR3 region of the VL (Fig. 2a, compared to NOL48-1-D5) caused this difference. The amino acid histidine is replaced by asparagine (H96N), which is a change from a basic amino acid to a polar amino acid. We could not determine a specific reason for the instability caused by this amino acid substitution at this position, but in general it has been reviewed by Rabia et al.^42^ that mutations that increase the affinity often result in decrease of stability. The lowest affinity/stability trade-off is described for mutations in the CDR3 region of the VH^42^. This is in line with our second top candidate NOL226-2-D10, which has two amino acid substitutions (A97V and F100L), both located in the CDR3 region of the VH (Fig. 2b, compared to NOL162-1-F5). This antibody shows no signs of instable behavior.

Previous studies have put forward the idea that during natural affinity maturation not only somatic hypermutations occur that are improving the affinity but also others that are compensating destabilizing effects^43–46^. If possible, a co-selection for affinity and stability during in vitro affinity maturation is recommended^42,47^. In our case, selection of antibodies was performed in scFv format while the final format with stability problems was the eqIgG6 format. Due to the format change at a later time point, when the antibody may have different properties, we recommend to always consider several candidates for the next step during the selection process of a final candidate.

Besides addressing IL-5, other targets for treatment of equine IBH with monoclonal antibodies could be conceivable. One alternative target could be the IL-5 receptor alpha subunit which is also addressed by the approved antibody Benralizumab for humans^16^. Other possible options could be targeting the eosinophil chemoattractant eotaxin-1 to avoid recruitment of eosinophil cells to the allergic site^48^ or IL-31 to reduce the allergic pruritus^49^. However, in this work we have focused on IL-5 as target since it is known as main activator and regulator of eosinophil cells and has the advantage of being only little involved in other immunological pathways, thus allowing a rather specific therapy^50^. Also, monoclonal antibodies against IL-5 in humans have not shown any significant safety concerns^16^. Eventually, only a clinical trial will provide a full evaluation of the therapeutic benefit of targeting IL-5 with monoclonal antibodies in the horse. Addressing other cytokines or chemokines that are involved in the molecular pathway of equine IBH (e.g. IL-4 and IL-13 for B cell class switch) could lead to a broader less specific immune reaction and a potential therapy against IgE, like Omalizumab for humans, could result in an increased risk for parasite infections.

There are many aspects to consider when developing therapeutic antibodies. Important requirements are the safety profile, efficacy, developability and biophysical parameters such as purity, stability, solubility and specificity^51,52^. During our selection process, we mainly focused on the inhibition effect of the antibody (IC_50_) and also took the binding activity (EC_50_) and producibility into account. In addition, the antibody stability and specificity were essential properties. In this regard, the final candidate NOL226-2-D10 features a strong inhibition effect (IC_50_ = 4 nM, in competitive assay IC_50_ = 9 nM), a nanomolar binding activity to the target (EC_50_ = 8.8 nM), satisfactory producibility in the transient expression system (~ 70 mg/L), stable behavior and no indication for unspecificity. The potential of NOL226-2-D10 to inhibit the binding of eqIL-5 to its receptor also in a competitive inhibition assay setup shows that the interaction of eqIL-5 to NOL226-2-D10 is more efficient than to the eqIL-5 receptor, which supports the eligibility of this antibody as candidate for IBH treatment.

Regarding the producibility, transient expression in Expi293F suspension cells does not provide a sufficient amount of protein for production of a therapeutic antibody. This can be solved by developing a stable CHO cell line for production^53^. An alternative solution would be the switch to a different production system in order to receive high yields at low cost. Possible alternative production systems could be based on plants^54^ or diatoms^55^, yeast cells^56^ or insect cells^57^.

In conclusion, we successfully selected a highly promising candidate that meets the commonly approved criteria of therapeutic antibodies regarding inhibition efficacy and stability. Additionally, the use of the eqIgG6 heavy chain could represent a new concept for the development of therapeutic antibodies against allergic diseases in the horse, as this isotype does not interact with the cellular components of the immune system and thus does not further amplify the immune reaction^20^. This concept will be further evaluated in in vivo studies for the treatment of equine IBH.

## Methods

### Design of expression vectors for production in Expi293F suspension cells

For eqIL-5 production, the corresponding gene was ordered without the signal peptide but with an 8 × His-tag (Uniprot primary accession eqIL-5: O02699). For eqIL-5-His, the gene was cloned into the pCSE2.7-hFc-XP vector via *Nco*I*/Xba*I (NEB). By using *Xba*I the Fc-part is cut out of the vector. For eqIL5-hFc, the gene without 8 × His-tag was cloned into the pCSE2.7-hFc-XP vector via *Nco*I*/Nhe*I (NEB). For scFv-hFc production, the scFv genes were subcloned into the pCSE2.7-hFc-XP vector via *Nco*I*/Not*I (NEB). For production of eqIgG6 antibodies, the backbone vectors were constructed by replacing the constant human domains of the vectors pCSEHh1c-XP (heavy chain) and pCSL3hl-XP/pCSL3hk-XP (light chain lambda/kappa) with equine domains. For this, the genes for IGHC6 (IMGT accession number IMGT000040), IGLC7^28^ and IGKC (IMGT accession number IMGT000053) were ordered and cloned into the human vector pCSEHh1c-XP via *BssH*II*/Xba*I (NEB) and into pCSL3hl-XP/pCSL3hk via *Age*I*/Xba*I (NEB) respectively, resulting in the equine vectors pCSEHeq6-XP, pCSLeq7l-XP and pCSLeqk-XP. These vectors were used for the subcloning of VH and VL via Golden Gate Assembly^58^ with the *Esp3*I restriction enzyme (NEB) and T4 DNA ligase (NEB). Genes for the expression of eqIL-5 receptor subunits were ordered without signal peptide (Uniprot primary accession Interleukin 5 receptor subunit A: A0A3Q2L5Z7; Uniprot primary accession Colony stimulation factor 2 receptor subunit beta: F7DHE0) and cloned via *Nco*I*/Xba*I (NEB) into the expression vector pCSE2.7-hFc-XP.

### Production of antigens and antibodies in Expi293F suspension cells

The antigen eqIL-5 and all antibodies were produced in Expi293F suspension cells (Thermo Fisher Scientific). The cells were cultivated in an incubation shaker (Minitron, Infors, 50 mm shaking stroke) at 37 °C, 110 rpm and 5% CO_2_ in Gibco FreeStyle F17 expression media (Thermo Fisher Scientific) supplemented with 8 mM L-Glutamine and 0.1% Pluronic F68 (PAN Biotech GmbH). The production scale was adjusted depending on the required protein amount and ranged between 10 and 250 mL (final volume after feeding). For transfection, cell density was between 1.5 × 10^6^ and 2.0 × 10^6^ cells/mL. 1 µg DNA/mL transfection volume and 5 µg 40 kDa PEI MAX (Polysciences)/mL transfection volume were first diluted separately in 5% of transfection volume, then mixed and incubated for 25 min at room temperature (RT) before adding to the cells. After 48 h incubation time, the cell culture volume was doubled by feeding with HyClone SFM4Trans-293 media (GE Healthcare) supplemented with 8 mM L-Glutamine and additionally 10% HyClone CellBoost5 (CN-F) Supplement (GE Healthcare) in relation to the final volume was added. After further five days of incubation, the cells were harvested via two centrifugation steps (first 4 min at 180 × *g* and 4 °C, then 20 min at 6969 × *g* and 4 °C). Subsequently, the supernatant was filtered for purification with a pore size of 0.2 µm.

### Protein purification

Proteins were purified depending on the production scale and tag. For His-tag purification, the protein was bound by nickel-loaded Sepharose (GE Healthcare), eluted with 250 mM Imidazole and dialyzed in 1 × PBS. For small scale production (10 mL) of scFv-hFc antibodies, MabSelect SuRe Protein A resin (Cytivia) was used. Buffer changing was done by Zeba Spin Desalting Columns (Thermo Fisher Scientific). For larger scale production of eqIgG6 antibodies, 0.4 mL HiTrap Fibro PrismA (Cytivia), 1 mL HiTrap Protein G HP (Cytivia) and HiPrep 26/10 Desalting (Cytivia) columns were used in the ÄKTA go (Cytivia), ÄKTA pure (Cytivia) or Profinia system (Bio-Rad). Here, eqIgG6 antibodies with a VH3 domain could be purified via the HiTrap Fibro PrismA (Cytivia) column, all other eqIgG6 antibodies were purified via the Protein G HP (Cytivia) column. All purifications were performed according to the manufacturers’ instructions.

### SDS-PAGE

Protein samples were diluted in 1 × PBS and 5 × Laemmli buffer with or without beta-mercaptoethanol (reducing/non-reducing) and boiled for 5 min at 95 °C (reducing) or 10 min at 56 °C (non-reducing). Then, 1 µg of protein was applied on a 15% SDS-PAGE and run for 1 h at 180 V, followed by staining with Coomassie Brilliant Blue and unstaining with 10% acetic acid.

### Antibody selection via phage display

Antibody selection via phage display was performed as described previously with some modifications^26,59^. Briefly, for panning in MTP, 2 µg antigen in 1 × PBS was immobilized in one well of a high binding 96-well MTP (Corning) at 4 °C overnight. The next day, wells were blocked for 1 h at RT with 320 µL 2% MPBST (2% (w/v) milk powder in 1 × PBS; 0.05% Tween20) and washed three times with H_2_O-Tween (H_2_O; 0.05% Tween20). In the meantime, 1 × 10^11^ colony forming units (cfu) of the libraries HAL9 (lambda) and HAL10 (kappa) were diluted in blocking solution (1% (w/v) milk powder and 1% (w/v) BSA in 1 × PBS; 0.05% Tween20) and pre-incubated on coated blocking solution for 1 h at RT. The libraries were then transferred into the antigen-coated wells and incubated for 2 h at RT. Next, unbound phage were removed by 10 × stringent washing with H_2_O-Tween and bound phage were eluted with 150 µL trypsin (10 µg/mL) for 30 min at 37 °C. The eluted phage solution was transferred to a polypropylene 96-deep-well plate (Greiner Bio-One) and incubated with 150 μL *E. coli* TG1 (OD_600nm_ = 0.5) first for 30 min at 37 °C followed by 30 min at 37 °C and 650 rpm in a MTP shaker (Vortemp 56, Labnet International). Next, 1 mL 2 × YT-GA (1.6% (w/v) tryptone; 1% (w/v) yeast extract; 0.5% (w/v) NaCl (pH 7.0); 100 mM D-Glucose; 100 μg/mL ampicillin) was added and *E. coli* was cultured for 1 h at 37 °C and 650 rpm. Then, 1 × 10^10^ cfu M13K07 helper phage was added and the culture was cultivated first for 30 min at 37 °C and then for 30 min at 37 °C and 650 rpm. To exchange the media, cells were centrifuged for 10 min at 3220 × *g*, the supernatant was discarded and the pellet was resuspended in 2 × YT-AK (1.6% (w/v) tryptone; 1% (w/v) yeast extract; 0.5% (w/v) NaCl (pH 7.0); 100 μg/mL ampicillin; 50 µg/mL kanamycin). Antibody phage were amplified overnight at 30 °C and 650 rpm. The next day, the culture was centrifuged for 10 min at 3220 × *g* and 50 µL of the supernatant, containing the amplified phage particles, was used for the next panning round.

For panning in solution, the antigen was biotinylated using the EZ-Link Sulfo-NHS-LC-Biotin kit (Thermo Fisher Scientific) according to the manufacturer’s protocol and dialyzed in 1 × PBS.

First, 1 × 10^11^ cfu of the libraries HAL9 (lambda) and HAL10 (kappa) were diluted in PBST (1 × PBS; 0.05% Tween20) and pre-incubated on coated 2% BSA ((w/v) in 1 × PBS) for 45 min at RT, followed by a second pre-incubation step with magnetic Streptavidin beads (Dynabeads M-280 Streptavidin, Invitrogen) in solution for 45 min rotating at RT. The supernatant containing the phage particles was separated using a magnetic stand and then mixed with 100 ng biotinylated antigen for 2 h under rotating conditions. Bound phage were captured by adding magnetic streptavidin beads for 25 min and then unbound phage were removed by washing the beads 10 × with PBST. From the elution step onwards, the protocol for panning in MTP was continued.

For the capture panning, first 2 µg mouse anti human IgG (Fc-specific) antibody (MC002-M, Abcalis GmbH) in 1 × PBS was immobilized in one well of a high binding 96-well MTP (Corning) for 1.5 h at RT. Wells were then blocked with 320 µL MPBST at 4 °C overnight and washed three times with H_2_O-Tween before adding 1 µg of hFc-tagged antigen in 1 × PBS for 1 h at RT and washing three times again. In the meantime, 1 × 10^11^ cfu of the libraries HAL9 (lambda) and HAL10 (kappa) were diluted in blocking solution and first pre-incubated on coated human IgG1 Fc-part (hFc) (AG002-hFc, Abcalis GmbH) for 1 h at RT and then pre-incubated on coated mouse anti human IgG (Fc-specific) antibody (MC002-M, Abcalis GmbH) for 1 h at RT. From the panning step onwards, the protocol for panning in MTP was continued.

For all three techniques, three or four panning rounds were performed with increasing stringency of the washing step to remove unbound phage (20 × in second panning round, 30 × in third and fourth panning round). The eluted phage after panning round three or four were titrated and single clones were selected for screening of monoclonal binders.

### Screening of monoclonal recombinant binders using E. coli scFv supernatant

Soluble scFvs with a C-terminal Myc-tag and 6 × His-tag were produced in *E. coli* in polypropylene 96-well MTPs (Greiner Bio-One) as described previously with some modifications^59^. Briefly, in each well 150 µL 2 × YT-GA media was inoculated with a single clone containing the scFv phagemid. The MTPs were incubated overnight at 37 °C and 800 rpm in a MTP shaker (Vortemp 56, Labnet International). The next day, 160 µL 2 × YT-GA media was inoculated with 10 µL of the overnight culture and incubated for 2 h at 37 °C and 800 rpm to reach an OD_600nm_ ~ 0.5. To induce the production of the scFv antibodies, cells were centrifuged for 10 min at 3220 × *g* and the pellets were resuspended in 160 µL 2 × YT-A supplemented with 50 µM isopropyl-β-D-thiogalactopyranoside (IPTG) and incubated overnight at 30 °C and 800 rpm. Then, cells were centrifuged for 15 min at 3220 × *g* and the scFv containing supernatant was used for the screening ELISA.

For the screening ELISA, 100 ng/well antigen in 1 × PBS was immobilized in a high binding 96-well MTP (Corning) overnight at 4 °C. As negative control for unspecific binding 1% BSA ((w/v) in 1 × PBS) was used. Wells were blocked with 320 µL 2% MPBST for 1 h at RT and washed three times with H_2_O-Tween. 40 µL of scFv containing supernatant from *E. coli* production was mixed with 60 µL 2% MPBST and incubated in the antigen-coated wells for 1.5 h at RT. Bound scFvs were detected using Hypermyc antibody conjugated with horseradish peroxidase (HRP) (AB-Hypermyc-M-HRP, Abcalis GmbH; diluted 1:30,000 in 2% MPBST) which recognizes the C-terminal c-Myc-tag. Bound antibodies were visualized by adding tetramethylbenzidine (TMB) substrate (19 parts TMB solution A (30 mM potassium citrate; 1% (w/v) citric acid (pH 4.1)) and 1 part TMB solution B (10 mM TMB; 10% (v/v) acetone; 90% (v/v) ethanol; 80 mM H_2_O_2_ (30%)) were mixed). The reaction was stopped with 1 N H_2_SO_4_ and the absorbance was measured at 450 nm with a reference at 620 nm in an ELISA plate reader (Epoch, BioTek). Monoclonal binders with a OD_450nm-620 nm_ > 0.1 and a signal/noise (antigen/BSA) ratio > 5 were sequenced and analyzed using VBASE2 (www.vbase2.org)^60^.

### Cellular in vitro inhibition assay

**Figure 4:**
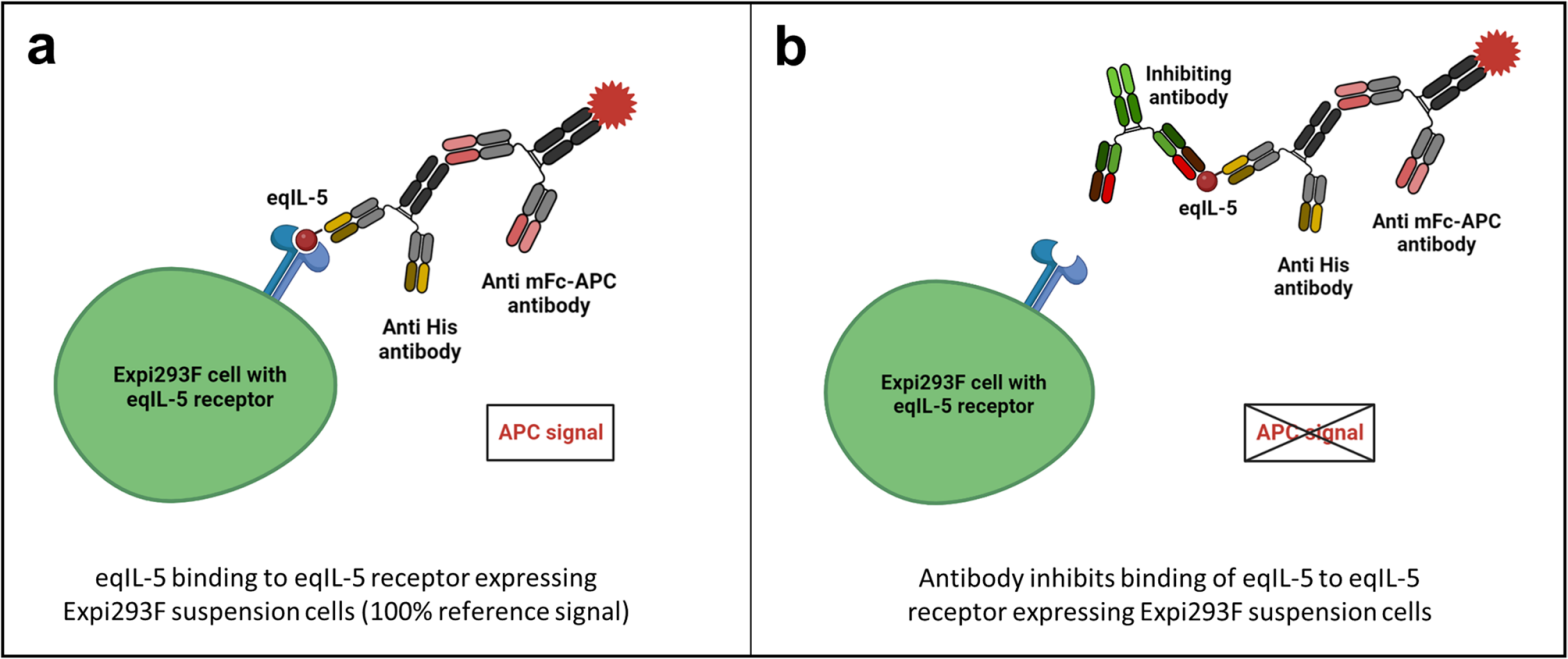
Setup of cellular in vitro inhibition assay. (**a**) EqIL-5 binding to eqIL-5 receptor expressing Expi293F suspension cells. The binding is detected via Penta-His antibody and a goat anti mouse Fc antibody coupled to APC. The APC signal represents the 100% binding reference signal. (**b**) Pre-incubation of antibody and antigen leads to inhibition of eqIL-5 binding to eqIL-5 receptor expressing Expi293 suspension cells. The APC signal is reduced compared to the 100% binding reference depending on the inhibition effect of the antibody.

Expi293F suspension cells were co-transfected according to the protocol above (“Production of antigens and antibodies in Expi293F suspension cells”) with the two receptor subunits in a molar ratio 1:1 and 5% enhanced green fluorescent protein (GFP) plasmid. 48 h after transfection, pre-incubated antigen and antibody were added to 5 × 10^5^ cells/well for 45 min on ice. In the screening, 1000 nM antibody and 5 nM eqIL-5 (related to dimer) were used (molar ratio 200:1). For the titration, 1000 nM–0.32 nM antibody and 5 nM eqIL-5 (related to dimer) were applied (molar ratio 200:1–0.064:1). All dilution and washing steps (centrifugation for 4 min at 280 × *g* and 4 °C) were performed with FACS buffer (2% FCS and 5 mM EDTA in 1 × PBS). Bound antigen was detected using a Penta-His antibody (3460, Qiagen; diluted 1:50) and a goat anti mouse Fc antibody coupled to APC (115-136-071, Jackson Immuno Research; diluted 1:50). Cells were analyzed in the flow cytometer (MACS Quant, Miltenyi Biotec). The setup of the cellular inhibition assay is visualized in Fig. 4.

Alive, single and GFP^+^ cells were gated and their APC median signal was measured (Supplementary Fig. S2). Background signal of the detection antibodies (no eqIL-5 and no test antibody applied) was subtracted from all signals. The binding signal of the interleukin (no test antibody applied) was set as 100% reference (Fig. 4a). Pre-incubation of antigen and tested antibody reduced the APC signal depending on the antibody’s inhibition effect (Fig. 4b). Data were analyzed with OriginPro using the Logistic5 Fit. IC_50_ values were calculated as the antibody concentration necessary to reduce the relative binding to 50%.

The antibodies NOL226-2-D10 and NOL162-1-F5 were additionally tested in a competitive cellular in vitro inhibition assay. This assay was performed as described above with the modification that antibody and antigen were not pre-incubated before adding them to the cells. Instead, 1000 nM–0.1 nM antibody was first titrated on 5 × 10^5^ eqIL-5 receptor expressing cells/well. Afterwards, 5 nM eqIL-5 (related to dimer) was applied (molar ratio 200:1–0.02:1) to the cell/antibody mixture for 45 min on ice. Then, the detection was performed as before using the Penta-His antibody (3460, Qiagen; diluted 1:50) and a goat anti mouse Fc antibody coupled to APC (115-136-071, Jackson Immuno Research; diluted 1:50).

For selected lead candidates an ELISA was performed to confirm that the APC signal reduction resulted due to the inhibition effect of the antibody and not due to steric hindrance of the anti His detection antibody. For this, 100 ng eqIL-5/well was immobilized in a high binding 96-well MTP (Corning) overnight at 4 °C. Wells were blocked with 2% MPBST for 1 h at RT and washed three times with H_2_O-Tween. Then, inhibiting antibody was titrated from 316 nM–0.001 nM diluted in 2% MPBST and incubated for 1 h at RT. As positive control Penta-His antibody (3460, Qiagen) was applied. Bound antibodies were detected with an HRP-conjugated anti polyhistidine antibody (A7058, Sigma; diluted 1:50,000 in 2% MPBST). The colorimetric reaction to visualize bound antibodies was performed as described above with TMB substrate and stopped with 1 N H_2_SO_4_. Absorbance was measured at 450 nm with a reference at 620 nm in an ELISA plate reader (Epoch, BioTek). Antibodies, that are not blocking the 8 × His-tag, are expected to lead to a constant signal independent of the antibody concentration.

### Titration ELISA

To test binding of equine antibodies to the eqIL-5 antigen, 100 ng eqIL-5/well was immobilized in a high binding 96-well MTP (Corning) overnight at 4 °C. Wells were blocked with 2% MPBST for 1 h at RT and washed three times with H_2_O-Tween. Then, antibodies were titrated from 316 nM to 0.001 nM diluted in 2% MPBST and incubated for 1 h at RT. Bound antibodies were detected with a goat IgG anti horse IgG (Fc-)HRP antibody (108-035-008, Jackson Immuno Research; diluted 1:1000 in 2% MPBST or SAB3700145, Sigma-Aldrich; diluted 1:5000 in 2% MPBST). The colorimetric reaction to visualize bound antibodies was performed as described above with TMB substrate and stopped with 1 N H_2_SO_4_. Absorbance was measured at 450 nm with a reference at 620 nm in an ELISA plate reader (Epoch, BioTek). Data were analyzed with OriginPro using the Logistic5 Fit. The EC_50_ values, which are defined as the antibody concentration at the half-maximum binding signal, were calculated for the comparison of the antibodies’ binding activity.

For comparison of NOL46-1-A1 and NOL46-1-E4 as scFv-eqFc and eqIgG, a second ELISA setup was performed to exclude a signal bias due to potential different binding of the detection antibody to the equine Fc-part in the two antibody formats. Here, 240 ng scFv-eqFc/well or 300 ng eqIgG/well of the antibodies NOL46-1-A1 and NOL46-1-E4 was immobilized in a high binding 96-well MTP (Corning) overnight at 4 °C. Wells were blocked with 2% MPBST for 1 h at RT and washed three times with H_2_O-Tween. Then, eqIL-5 was titrated from 100 nM to 0.001 nM diluted in 2% MPBST and incubated for 1 h at RT. Bound antibodies were detected with an anti polyhistidine-HRP antibody (A7058, Sigma-Aldrich; diluted 1:50,000 in 2% MPBST). Bound antigen was visualized as described above by adding TMB substrate and the reaction was stopped with 1 N H_2_SO_4_. The absorbance was measured at 450 nm with a reference at 620 nm in an ELISA plate reader (Epoch, BioTek). Data were analyzed with OriginPro using the Logistic5 Fit.

### Stability assays

Selected antibodies (NOL48-1-D5 and NOL162-1-F5) were tested regarding their stability in titration ELISA, cellular inhibition assay and SEC. A timeline for these assays is shown in Supplementary Fig. S9.

For titration ELISA, the antibodies were stored at a concentration of 0.3 mg/mL for 0, 2, 7, 14 and 28 days at 37 °C and for 28 days at 4 °C. The titration ELISA was performed as described above (“Titration ELISA”). For the cellular inhibition assay, antibodies were stored at a concentration of 0.3 mg/mL for 0 and 28 days at 37 °C. A titration assay was performed as described above (“Cellular *in vitro* inhibition assay”). For SEC, the antibodies were stored at a concentration of 0.3 mg/mL for 0 and 28 days at 37 °C. Samples were diluted with 150 nM sodium phosphate buffer to a concentration of 25 µg/mL and were sterile-filtered with a pore size of 0.2 µm. Measurements were performed with the Chromaster HPLC system (Hitachi) using the AdvanceBio SEC 300A 2.7 µm 4.6 × 300 mm column (Agilent Technologies) according to the manufacturer’s instruction.

### Long-term stability assays

The antibody NOL226-2-D10 was tested regarding its long-term stability in titration ELISA, cellular inhibition assay and SEC. Assays were performed as described above (“Stability assays”) with a modification of the storage conditions. For titration ELISA and cellular inhibition assay, the antibody was stored at a concentration of 1.86 mg/mL for 0, 2 and 6 months at 4 °C. For SEC, the antibody was stored at a concentration of 1.86 mg/mL for 1 months at 4 °C and for 2 months at 21 °C.

### Unspecificity ELISA

The antigens eqIL-5, 1 × PBS, BSA, DNA, lysozyme, LPS, Expi293F suspension cell lysate and unrelated scFv-His antibody were immobilized in a high binding 96-well MTP (Corning) with 10 µg/mL diluted in 1 × PBS for 3 h at RT and blocked with 2% MPBST overnight at 4 °C. After washing three times with H_2_O-Tween, 100 nM of the antibodies to be tested was added to the wells and incubated for 1 h at RT. The detection was performed with a goat IgG anti horse IgG (Fc-)HRP antibody (108-035-008, Jackson Immuno Research; diluted 1:1000 in 2% MPBST). The colorimetric reaction to visualize bound antibodies was performed as described above with TMB substrate and stopped with 1 N H_2_SO_4_. Absorbance was measured at 450 nm with a reference at 620 nm in an ELISA plate reader (Epoch, BioTek). The background signal, when only antigen and detection antibody were applied, was subtracted for the calculation of the ΔOD_450nm−620 nm_.

### In vitro affinity maturation

**Figure 5:**
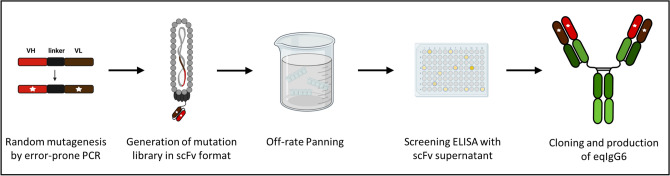
Process of in vitro affinity maturation.

The in vitro affinity maturation was performed as described previously in detail with some modifications^61^. Figure 5 illustrates the process.

Briefly, three consecutive rounds of error prone PCR with the parental scFv gene were performed to insert random nucleotide mutations. For this, the GeneMorphII Random Mutagenesis Kit (Agilent Technologies) was used according to the manufacturer’s instructions (in the first round 10 ng phagemid as template DNA, in the second and third round 2 ng PCR product of the previous round as template DNA; 30 PCR cycles). For the library construction, the mutated scFv genes were cloned into the pHAL30 vector via *Nco*I*/Not*I (NEB) cloning and electrocompetent *E. coli* ER2738 cells (Lucigen) were transformed with the library phagemid DNA. The *E. coli* cells were then used for packaging of the phagemids into phage particles. 2 × YT-GA media was inoculated with *E. coli* and cells were incubated until OD_600_ ~ 0.5. Then, 20 mL of the culture was infected with 2.5 × 10^11^ cfu M13K07 helperphage. After media exchange to 2 × YT-AK, a 50 mL culture was cultivated for 24 h at 30 °C and 210 rpm in an incubation shaker for the phage production. The next day, the phage containing supernatant was separated from the bacteria (30 min, 12,000xg, 4 °C) and phage particles were precipitated with PEG/NaCl (2.5 M NaCl; 20% (w/v) PEG 6000) (20% of final volume) overnight at 4 °C. Next, the phage suspension was centrifuged (1 h, 20,450 × *g*, 4 °C) and the phage pellet was resuspended in 10 mL phage dilution buffer (PDB) (10 mM Tris–HCl pH 7.5; 20 mM NaCl; 2 mM EDTA) and filtered with a pore size of 0.45 µm. A second precipitation with PEG/NaCl was performed overnight at 4 °C. Then, the phage suspension was centrifuged (30 min, 47,810 × *g*, 4 °C) and the pellet was resuspended in 1 mL PDB. The suspension was centrifuged again (1 min, 16,000 × *g*, RT) and the phage containing supernatant stored at 4 °C for further use. With this scFv mutagenesis library an off-rate panning was performed. In contrast to the panning described above (“Antibody selection via phage display”), here only one panning round was performed. 10 ng antigen in 1 × PBS was immobilized in one well of a MaxiSorp 8-well stripe (Thermo Fisher Scientific) at 4 °C overnight. Then, wells were blocked for 1 h at RT with 320 µL 2% MPBST and washed three times with H_2_O-Tween. In the meantime, 1 × 10^10^ cfu of the scFv mutagenesis libraries were diluted in blocking solution and first pre-incubated on coated blocking solution for 45 min at RT and then pre-incubated on coated unrelated scFv-His protein for 45 min at RT. The libraries were then transferred into the antigen-coated wells and incubated for 1 h at RT. Next, unbound phage were removed by 30 × stringent washing with H_2_O-Tween and then stripes were incubated up to 3 weeks at 4 °C in 1 × PBS under stirring conditions. Several washing steps (30 × stringent washing with H_2_O-Tween) were performed in between and phage were eluted at different time points (5 days, 7 days, 12 days, 14 days and 21 days) using 150 µL trypsin (10 µg/mL)/well for 30 min at 37 °C. Eluted phage were titrated and single clones were selected for screening of monoclonal binders. Screening was performed as described above (“Screening of monoclonal recombinant binders using *E. coli* scFv supernatant”). Now, the parental scFvs were tested in parallel as reference. Clones with significantly increased signals (> 1.3 × increased compared to the parental scFv signal) were selected and cloned into eqIgG6 format.

## Supplementary Information

Supplementary Figures.

## Data Availability

The authors declare that the data supporting the findings of this study are available within the paper and its supplementary information files. Primary data are available from the corresponding author on reasonable request.
