# Supplementary material for: Development of an inhibiting antibody against equine interleukin 5 to treat insect bite hypersensitivity of horses
Source: Sci Rep. 2023 Mar 10;13:4029. doi: 10.1038/s41598-023-31173-y (PMC10000358; doi:10.1038/s41598-023-31173-y)
Supplement: Supplementary file 1 — Supplementary Figures. [file 41598_2023_31173_MOESM1_ESM.pdf]

Supplementary Information

Development of an inhibiting antibody against equine interleukin 5 to treat insect bite hypersensitivity of horses

Nora Langreder[1], Dorina Schäckermann[1,4], Doris Meier[1], Marlies Becker[1], Maren Schubert[1], Stefan Dübel[1], Thomas Reinard[2], Stefanie Figge-Wegener[3], Kristine Roßbach[4], Wolfgang Bäumer[5], Simone Ladel[4], Michael Hust[1]

[1] Technische Universität Braunschweig, Institut für Biochemie, Biotechnologie und Bioinformatik, Spielmannstr. 7, 38106 Braunschweig, Germany

[2] Leibniz Universität Hannover, Institut für Pflanzengenetik Abt II, Herrenhäuser Straße 2 30419 Hannover, Germany

[3] Novihum Technologies GmbH, Weidenstraße 70-72, 44147 Dortmund, Germany

[4] Wirtschaftsgenossenschaft deutscher Tierärzte eG (WDT), Siemensstraße 14, 30827 Garbsen, Germany

[5] Freie Universität Berlin, Institut für Pharmakologie und Toxikologie, Fachbereich Veterinärmedizin, Koserstraße 20, 14195 Berlin, Germany

Corresponding author:
Michael Hust
Technische Universität Braunschweig
Institut für Biochemie, Biotechnologie und Bioinformatik
Spielmannstr. 7
38106 Braunschweig, Germany
E-Mail: m.hust@tu-bs.de

Supplementary Figure S1: SDS-PAGE of recombinantly produced eqIL-5

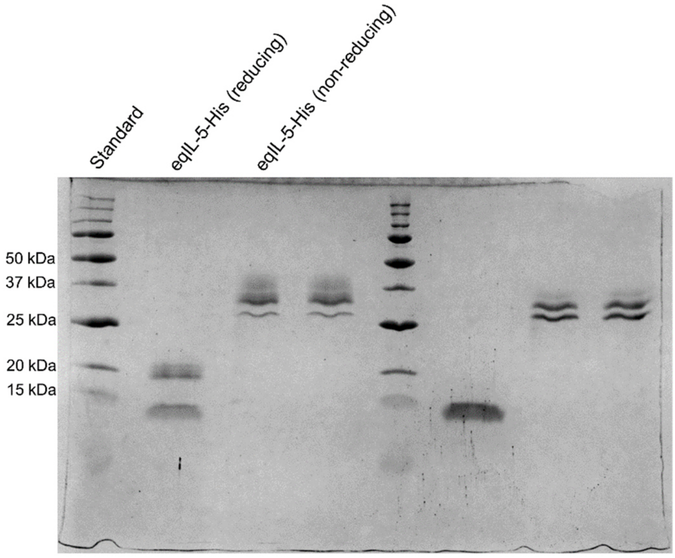

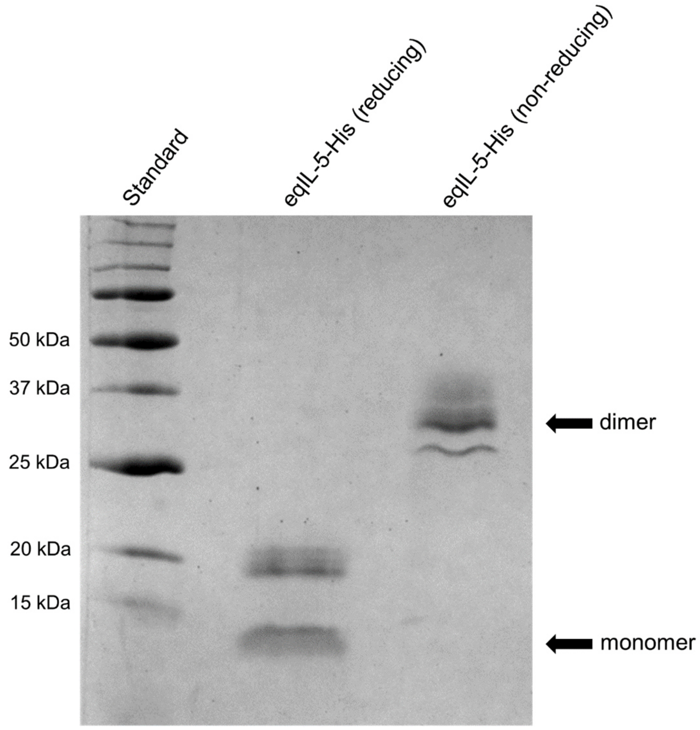

Supplementary Figure S1: SDS-PAGE of recombinantly produced eqIL-5. EqIL-5-His was produced recombinantly in Expi293F suspension cells and purified via His-tag. 1 µg of protein was applied on a 15% SDS-PAGE. SDS-PAGE was stained with Coomassie Brilliant Blue. The upper image shows the original gel with additional samples unrelated to this experiment. The lower image shows a cropped version of the original gel, only presenting the relevant samples for this experiment. The gel was not further modified. The lower image shows the purified eqIL-5-His under reducing conditions (left) and non-reducing conditions (right).

Under reducing conditions eqIL-5 forms a monomer with a double band at approximately 15/20 kDa. The additional 20 kDa band is expected due to N-glycosylation of the protein at the amino acid positions 76 and 90 (Uniprot O02699). Also, the commercial eqIL-5 (Bio-Techne GmbH) is described to run at around 19 kDa as a monomer. Under non-reducing conditions eqIL-5 forms a dimer with a band at approximately 30 kDa.

Supplementary Figure S2: Flow cytometry inhibition analysis

a **Gating strategy**

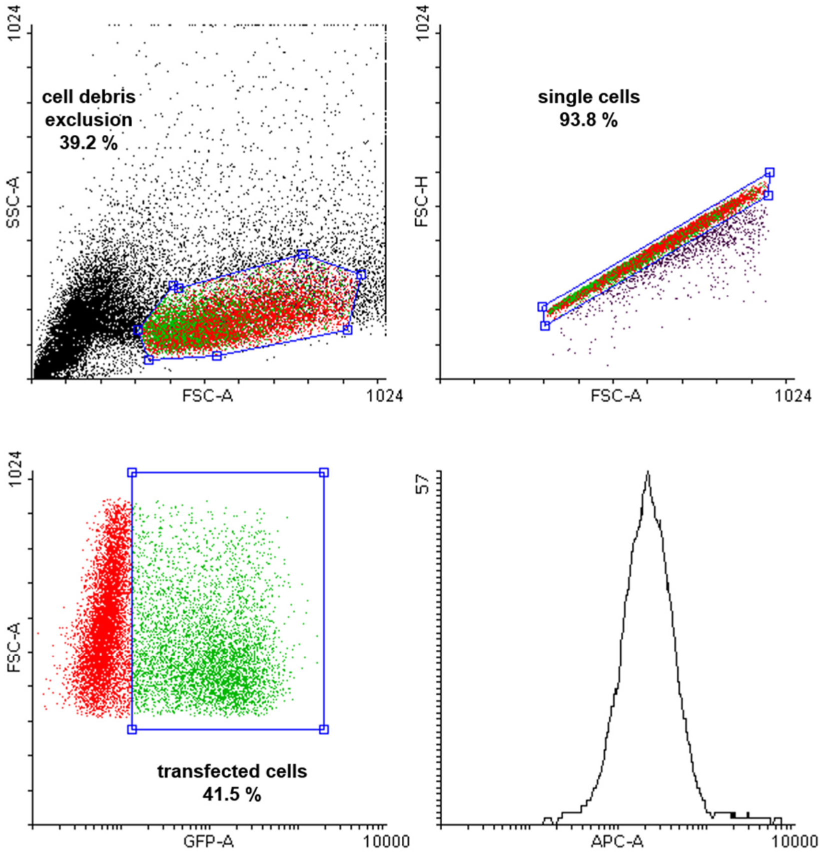

b **Analysis of eqIL-5 binding**

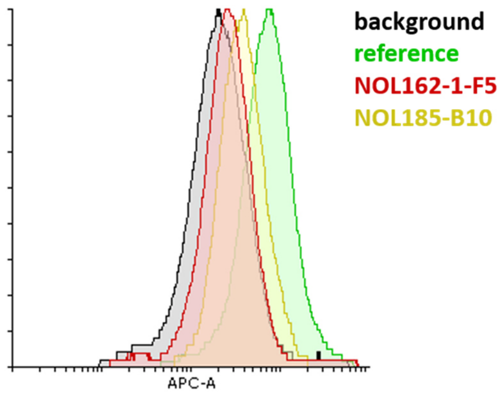

Supplementary Figure S2: Flow cytometry inhibition analysis. (a) Gating strategy. **(b)** Analysis of eqIL-5 (5 nM related to dimer) binding to living Expi293F suspension cells expressing eqIL-5 receptor blocked by 100 nM antibodies. The antibodies NOL162-1-F5 and NOL185-B10 were used as examples. The background control were transfected and stained cells without addition of eqIL-5 antigen and inhibiting antibody. The reference were transfected and stained cells incubated with eqIL-5 antigen but without addition of inhibiting antibody.

Supplementary Figure S3: Schematic illustration of antibodies in scFv-hFc, scFv-eqFc and eqIgG format

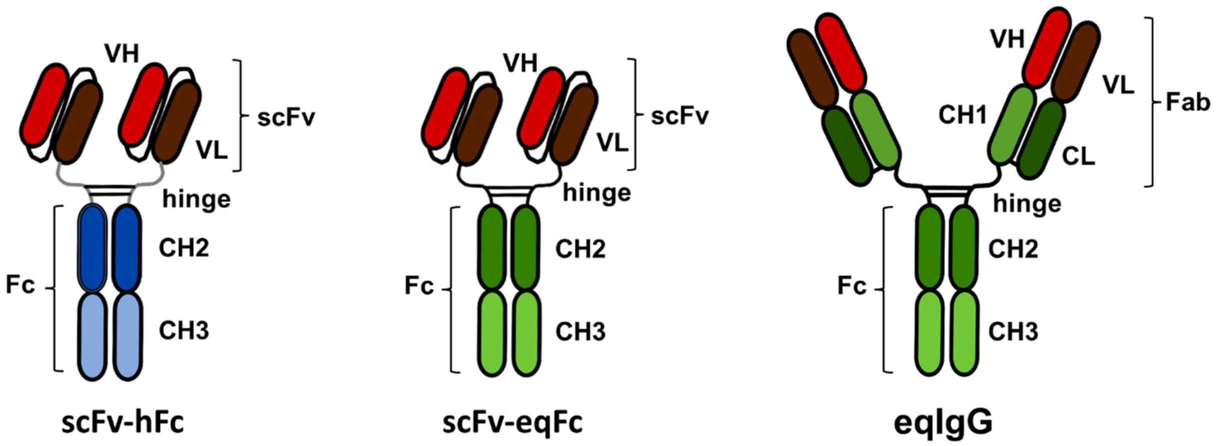

Supplementary Figure S3: Schematic illustration of antibodies in scFv-hFc, scFv-eqFc and eqIgG format. scFv-hFc: scFv-Fc with human Fc-part of human IgG1, scFv-eqFc: scFv-Fc with equine Fc-part of equine IgG6, eqIgG with equine Fc-part of equine IgG6. Human constant domains are indicated in blue, human hinge region in grey, equine constant domains in green, equine hinge region in black, human heavy chain variable domain (VH) in red, human light chain variable domain (VL) in brown.

Supplementary Figure S4: Comparison of antibody formats in the cellular inhibition assay and titration ELISA

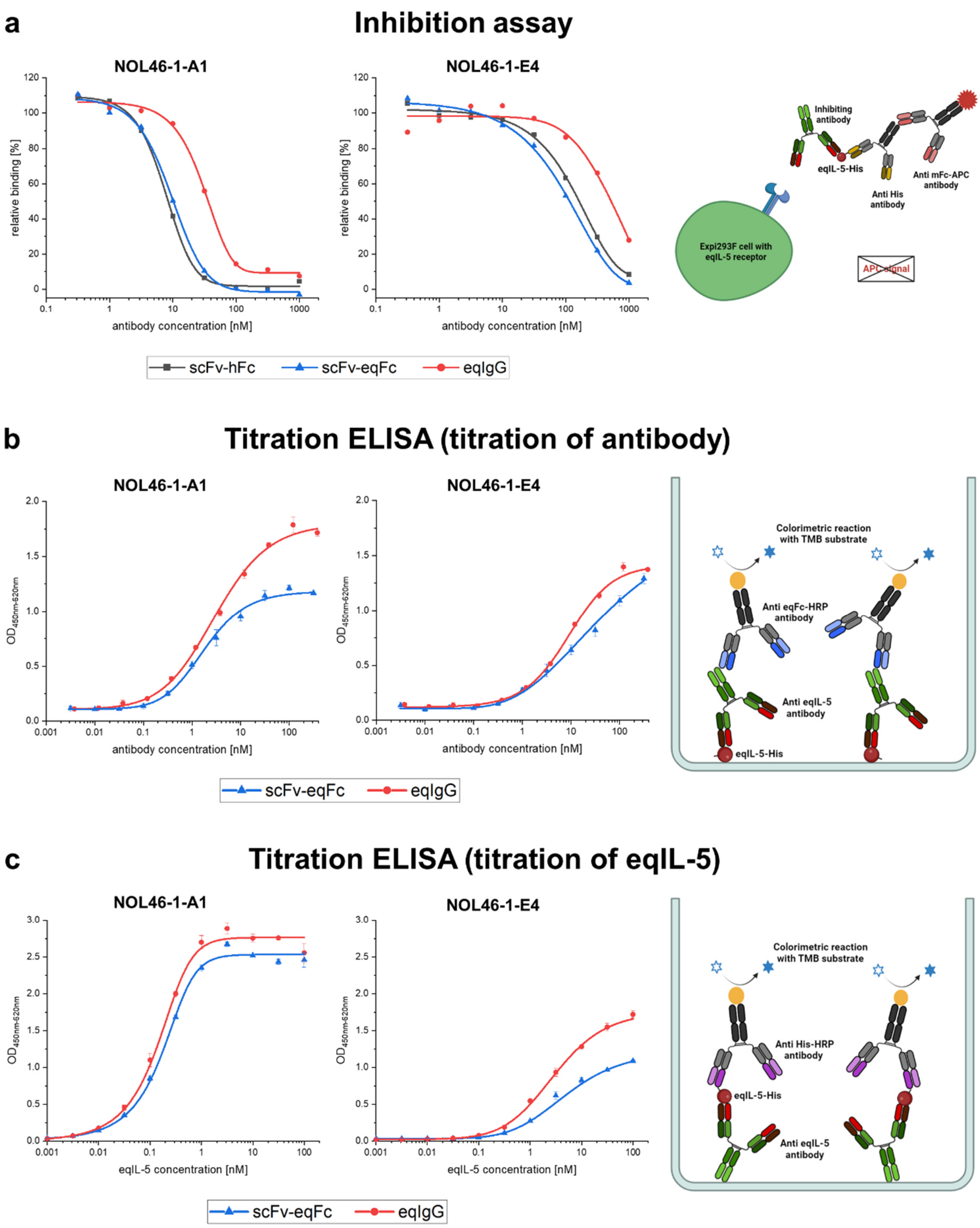

Supplementary Figure S4: Comparison of antibody formats in the cellular inhibition assay and titration ELISA. (a) Titration inhibition assay with the antibodies NOL46-1-A1 and NOL46-1-E4 in scFv-hFc, scFv-eqFc and eqIgG format using 1000 nM – 0.32 nM antibody and 5 nM antigen (related to dimer). Curves were determined with OriginPro using the Logistic5 Fit. Titration assays were performed in single measurements (n = 1). **(b)** Titration ELISA with the antibodies NOL46-1-A1 and NOL46-1-E4 in scFv-eqFc and eqIgG format. EqIL-5 was immobilized and the antibodies were titrated with 316 nM – 0.0036 nM. Curves were determined with OriginPro using the Logistic5 Fit. Measurements were performed in triplicates (n = 3). **(c)** Titration ELISA with the antibodies NOL46-1-A1 and NOL46-1-E4 in scFv-eqFc and eqIgG format. Antibodies were immobilized and eqIL-5 was titrated with 100 nM – 0.001 nM. Curves were determined with OriginPro using the Logistic5 Fit. Measurements were performed in triplicates (n = 3). The assay setups are demonstrated exemplarily with the eqIgG antibody.

Supplementary Figure S5: Stability assays with lead candidates

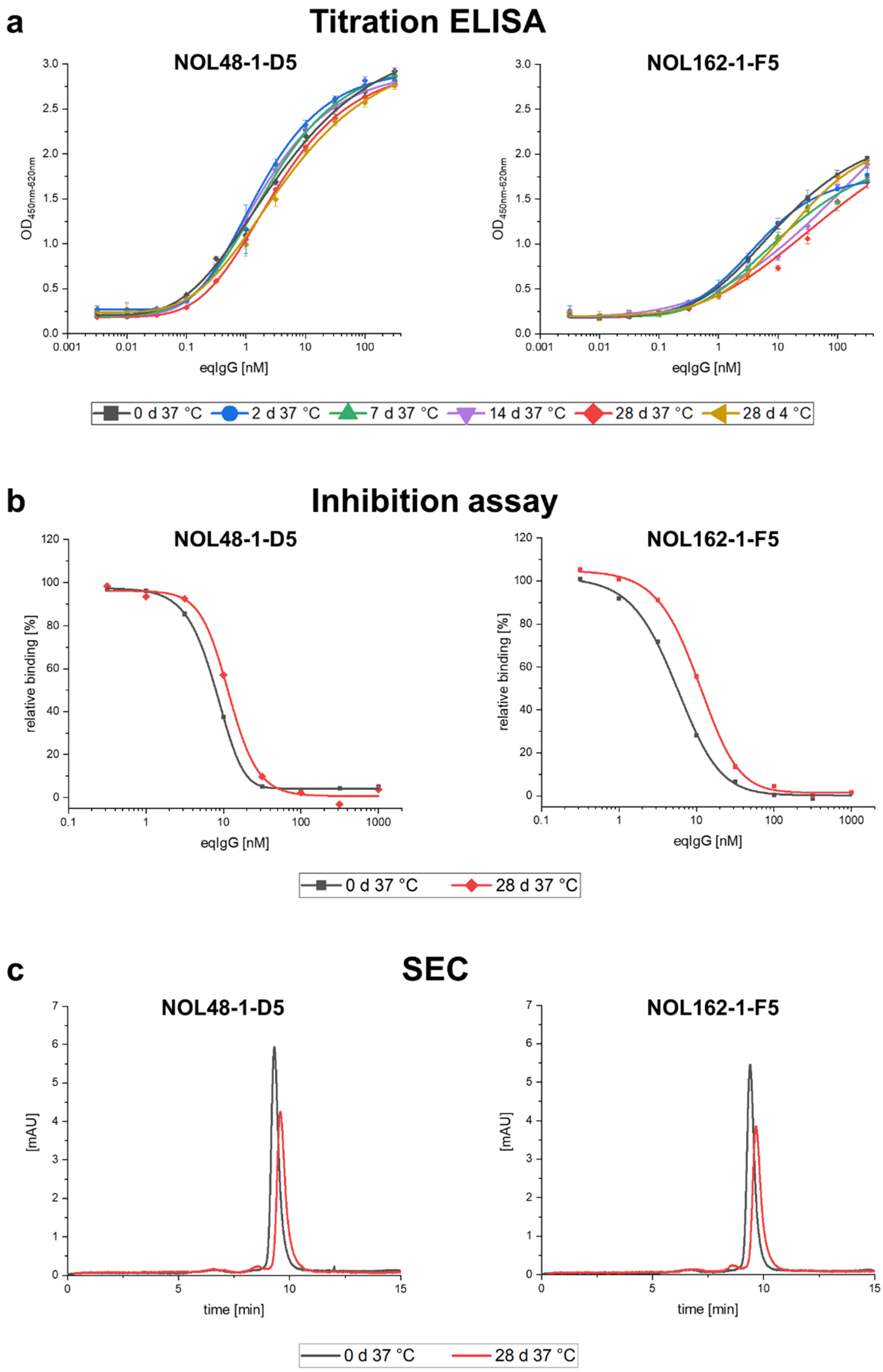

Supplementary Figure S5: Stability assays with lead candidates. (a) Titration ELISA with eqIgG6 antibodies NOL48-1-D5 and NOL162-1-F5 stored at 0.3 mg/mL for 0, 2, 7, 14 and 28 days at 37 °C and for 28 days at 4 °C. Curves were determined with OriginPro using the Logistic5 Fit. Measurements were performed in triplicates (n = 3). **(b)** Inhibition assay with eqIgG6 antibodies NOL48-1-D5 and NOL162-1-F5 stored at 0.3 mg/mL for 0 and 28 days at 37 °C. Curves were determined with OriginPro using the Logistic5 Fit. Measurements were performed in single measurements (n = 1). **(c)** SEC measurement with eqIgG6 antibodies NOL48-1-D5 and NOL162-1-F5 stored at 0.3 mg/mL for 0 and 28 days at 37 °C. Measurements were performed in duplicates (n = 2).

In the titration ELISA (Supplementary Fig. S5a), the antibody NOL48-1-D5 showed nearly no decrease of signal when stored up to 28 days at 37 °C or 4 °C. The antibody NOL162-1-F5 showed slight decrease of the signal over time. For this antibody the comparably low affinity in ELISA resulted in curves that sometimes did not reach complete saturation and could therefore not always be fit well with the Logistic5 fit. This makes the curves at the different time points less comparable. Despite this, the low affinity in ELISA is no criterion for exclusion since the main aspect is the functionality tested in the cellular inhibition assay. Overall, both antibodies displayed sufficient stability behavior.

In the inhibition assay (Supplementary Fig. S5b), both antibodies had a slight reduction in inhibition after being stored for 28 days at 37 °C. Nevertheless, this reduction is acceptable for the long storage time.

In the SEC (Supplementary Fig. S5c), both antibodies had their main peak as monomer before and after being stored for 28 days at 37 °C. A slight increase of a dimer peak left of the monomer peak was observed after 28 days at 37 °C for both antibodies, but overall, no significant hint for instability was measured. The shift of the monomer peak to the right arose since the samples stored for 28 days at 37 °C were run over the column a later time point.

Supplementary Figure S6: Unspecificity ELISA with lead candidates

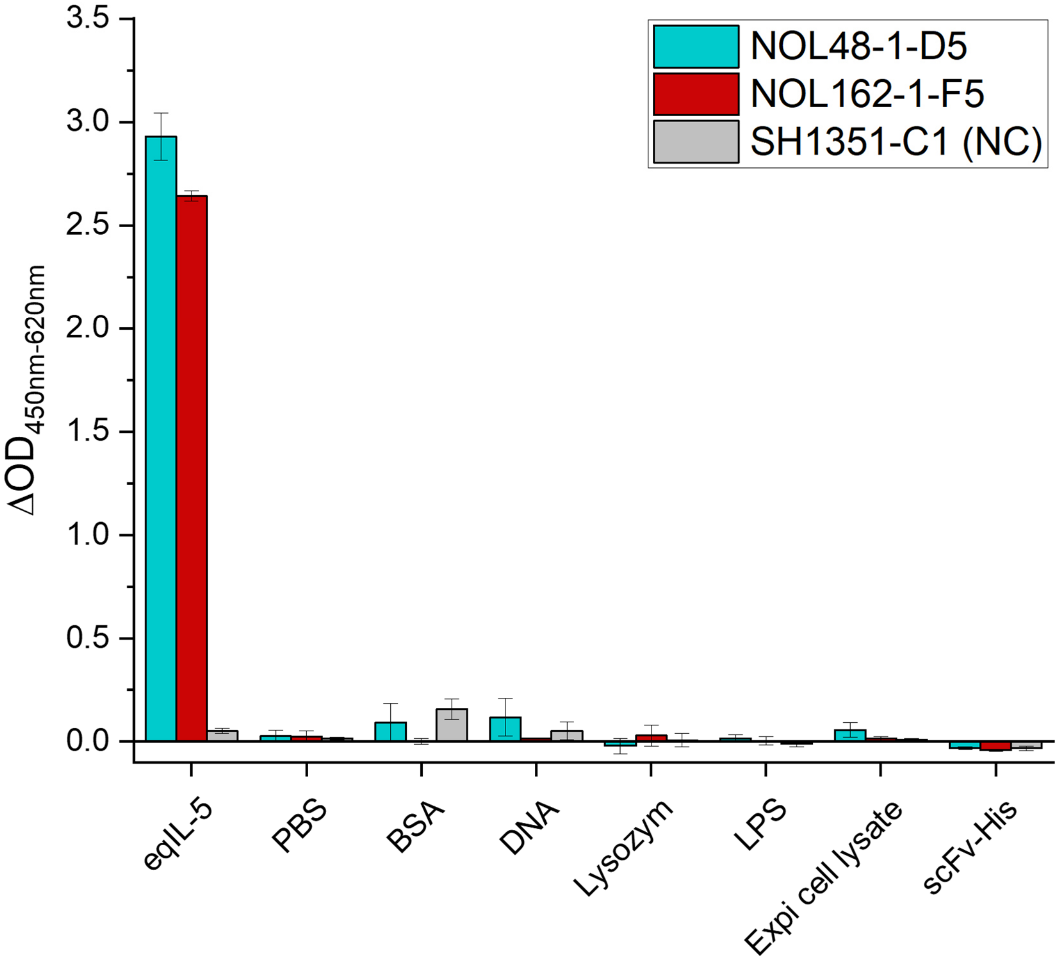

Supplementary Figure S6: Unspecificity ELISA with lead candidates. The eqIgG6 antibodies (100 nM) NOL48-1-D5, NOL162-1-F5 and the isotype control SH1351-C1 were tested on different antigens (10 µg/mL): eqIL-5-His, PBS, BSA, DNA, lysozyme, LPS, Expi293F suspension cell lysate, unrelated scFv-His antibody. The background signal, when only antigen and detection antibody were applied, was subtracted for the calculation of the $\Delta OD_{450nm\text{-}620nm}$. Measurements were performed in triplicates (n = 3).

In this assay, the antibodies NOL48-1-D5 and NOL162-1-F5 displayed no binding to any other antigen but eqIL-5.

Supplementary Figure S7: Characterization of NOL226-2-D10 in terms of stability and specificity

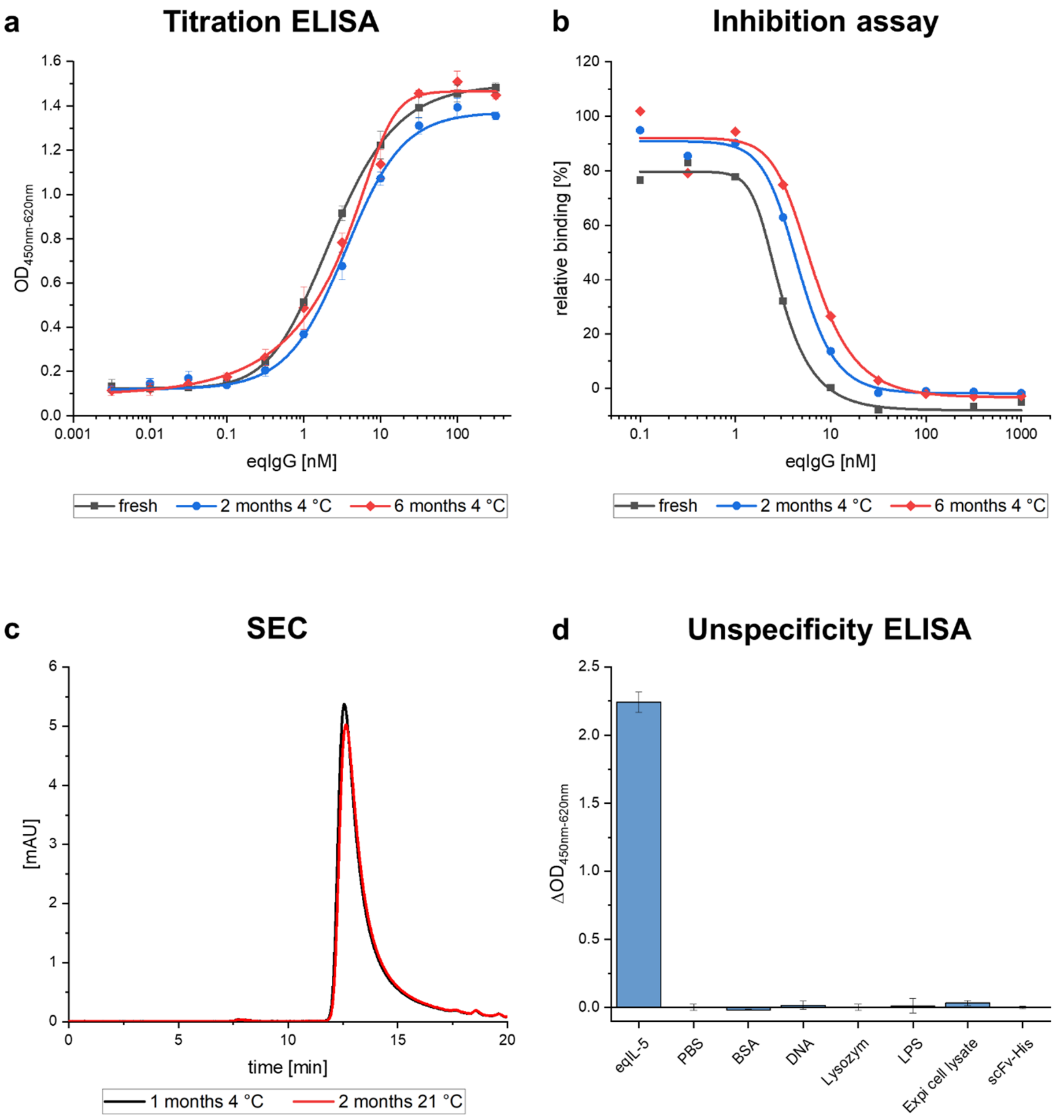

Supplementary Figure S7: Characterization of NOL226-2-D10 in terms of stability and specificity. (a) Titration ELISA with NOL226-2-D10 stored at 1.86 mg/mL for 0, 2 and 6 months at 4 °C. Curves were determined with OriginPro using the Logistic5 Fit. Measurements were performed in triplicates (n = 3). **(b)** Inhibition assay with NOL226-2-D10 stored at 1.86 mg/mL for 0, 2 and 6 months at 4 °C. Curves were determined with OriginPro using the Logistic5 Fit. Measurements were performed in single measurements (n = 1). **(c)** SEC measurement with NOL226-2-D10 stored at 1.86 mg/mL for 1 month at 4 °C and for 2 months at 21 °C. Measurements were performed in duplicates (n = 2). **(d)** Unspecificity ELISA with NOL226-2-D10 (100 nM) tested on different antigens (10 µg/mL): eqIL 5-His, PBS, BSA, DNA, lysozyme, LPS, Expi293F suspension cell lysate, unrelated scFv-His antibody. The background signal, when only antigen and detection antibody were applied, was subtracted for the calculation of the $\Delta OD_{450nm\ 620nm}$. Measurements were performed in triplicates (n = 3).

Supplementary Figure S8: Competitive cellular *in vitro* inhibition assay

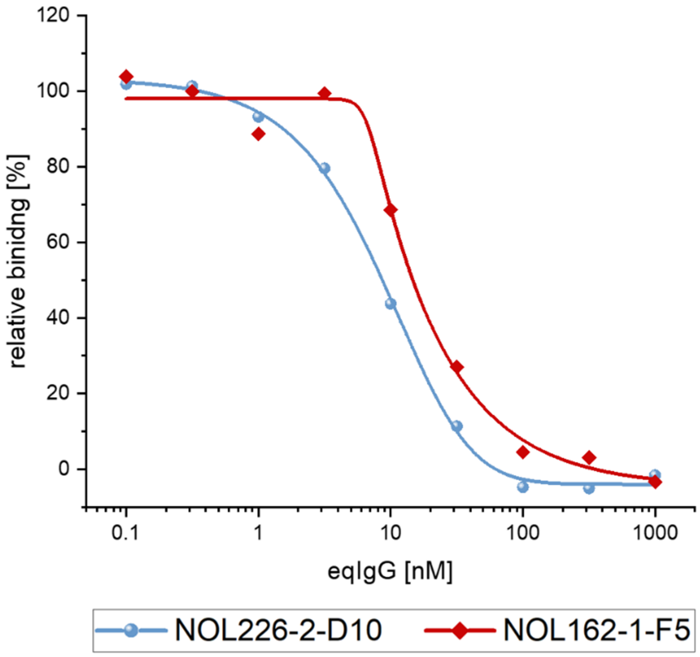

Supplementary Figure S8: Competitive cellular *in vitro* inhibition assay. The antibody NOL226-2-D10 and its parental antibody NOL162-1-F5 were titrated on eqIL-5 receptor expressing cells using 1000 nM – 0.1 nM antibody. Afterwards, 5 nM antigen (related to dimer) (molar ratio 200:1 – 0.02:1) was added. IC_{50} values were determined with OriginPro using the Logistic5 Fit. Measurements were performed in single measurements (n = 1).

Supplementary Figure S9: Timeline for stability assays

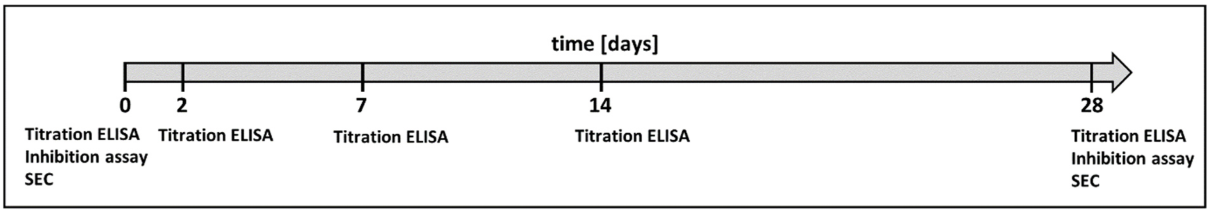

Supplementary Figure S9: Timeline for stability assays.
